# Supplementary material for: Estimation of SARS-CoV-2 mortality during the early stages of an epidemic: A modeling study in Hubei, China, and six regions in Europe
Source: PLoS Med. 2020 Jul 28;17(7):e1003189. doi: 10.1371/journal.pmed.1003189 (PMC7386608; doi:10.1371/journal.pmed.1003189)
Supplement: S1 Text — Further details about data sources, model, external validation, additional results, and sensitivity analyses. (PDF) [file pmed.1003189.s001.pdf]

Supplementary appendix to:
Estimation of SARS-CoV-2 mortality during the early stages of an epidemic: a modeling study in Hubei, China, and six regions in Europe

Anthony Hauser[a], Michel J. Counotte[a], Charles C. Margossian[b], Garyfallos Konstantinoudis[c], Nicola Low[a], Christian L. Althaus[a], and Julien Riou[a,d,*]

[a]Institute of Social and Preventive Medicine, University of Bern, Bern, Switzerland
[b]Department of Statistics, Columbia University, New York, NY
[c]MRC Centre for Environment and Health, Department of Epidemiology and Biostatistics, School of Public Health, Imperial College London, London, UK
[d]Division of infectious diseases, Federal Office of Public Health, Bern, Switzerland
[*]Corresponding author (julien.riou@ispm.unibe.ch)

July 10, 2020

Contents

1 Data **3**

2 Transmission model **8**
2.1 General structure . . . 8
2.2 Force of infection . . . 8
2.3 Incubation, infectiousness and symptom onset . . . 9
2.4 Proportion of symptomatic infections . . . 9
2.5 Contribution of presymptomatic transmission . . . 9
2.6 Control measures . . . 10

3 Model simulation **10**
3.1 Initial conditions and simulation settings . . . 10
3.2 Dummy compartments and outputs . . . 10
3.3 Delayed mortality . . . 11
3.4 Inference . . . 11
3.5 Estimates of mortality . . . 12
3.6 Implementation and diagnostics . . . 13

4 External validation **20**

5 Additional results **21**
5.1 Additional results in Hubei, China . . . 21
5.2 Additional results in Austria . . . 22
5.3 Additional results in Baden-Württemberg, Germany . . . 23
5.4 Additional results in Bavaria, Germany . . . 24
5.5 Additional results in Lombardy, Italy . . . 25
5.6 Additional results in Spain . . . 26
5.7 Additional results in Switzerland . . . 27

6 Sensitivity analyses **28**
6.1 Correction of cases and deaths in Hubei, China . . . 28
6.2 Lower susceptibility in 0-19 year olds . . . 29
6.3 Ascertainment in the highest age class . . . 30
6.4 Earlier date of analysis . . . 31
6.5 Lower contribution of presymptomatic transmission . . . 37
6.6 Reduced time between symptom onset and death . . . 39

7 Stan code **40**

1 Data

We distinguished between country-specific data and general data that are identical across regions. Table 1 summarizes the values and sources of the collected data.

Table 1: Summary of data sources and fixed parameters.

Symbol	Comment	Value	Unit	Source
Country-specific data				
$\mathbb{A}$	Confirmed cases of SARS-CoV-2 infection by day of symptom onset	see Fig S1-S7A	counts	see Table 2
$\mathbb{B}$	Age distribution of all confirmed cases	see Fig S1-S7B	counts	see Table 2
$\mathbb{C}$	Deaths among patients with confirmed SARS-CoV-2 infection by day	see Fig S1-S7C	counts	see Table 2
$\mathbb{D}$	Age distribution of the deaths among patients with confirmed SARS-CoV-2 infection	see Fig S1-S7B	counts	see Table 2
$\mathbb{E}$	Age distribution of the population	see Fig S1-S7B	counts	[1, 2]
$\mathbb{F}$	Matrix of daily numbers of contacts between each age class in Shanghai (used for Hubei) and in Europe (POLYMOD, used for the 6 European regions)	see Fig S1-S7D	per day	[3, 4]
$\mathbb{G}$	Proportion of confirmed cases that are reported by date of symptom onset	see Table 2	percentage	see Table 2
$\mathbb{H}$	Proportion of deaths that are reported by date of death	see Table 2	percentage	see Table 2
General data				
$\mathbb{I}$	Distribution of time from symptom onset to death	log-$\mathcal{N}(20.2, 11.6)$	days	[5]
$1/\tau_1 + 1/\tau_2$	Incubation period	5.0	days	[6, 7, 8, 9, 10]
g	Generation time	5.2	days	[6, 11]
q	Contribution of presymptomatic infections to transmission	46%	percentage	[9, 11, 12]
$1/\tau_2$	Duration of presymptomatic infectiousness	2.3	days	[12]
γ	Time from symptom onset to death (mean - standard deviation)	20.2 - 11.6	days	[13]

We modelled the dynamics of the SARS-CoV-2 epidemic in the following regions: Austria, Baden-Württemberg (Germany), Bavaria (Germany), Hubei (China), Lombardy (Italy), Switzerland and Spain. For each of these regions, we collected four types of data related to the SARS-CoV-2 epidemic: (1) the number of confirmed cases by day of symptom onset $\mathbb{A}$, (2) the age distribution of all confirmed cases $\mathbb{B}$, (3) the number of deaths among confirmed cases by day of death $\mathbb{C}$, and (4) the age distribution of all reported deaths $\mathbb{D}$. In Baden-Württemberg and Bavaria, both the distributions by age of cases and deaths were collected at the national level, as this information was not available at the regional level. In Lombardy, the age distribution of cases were collected at the regional level and the age distribution of deaths at the national level.

In some cases, counts of confirmed cases by date of symptom onset represented only a part of the total number of confirmed cases. In these cases, we accounted for the disparity by scaling the model output by the proportion of confirmed cases with available date of symptom onset $\mathbb{G}$, assuming that dates of disease onset were missing at random (section 3.2). When the same thing occurred for deaths (only in Hubei due to the later

correction in the number of SARS-CoV-2-related deaths), we used a similar approach with the scaling factor $\mathbb{H}$.

In addition, we also collected the age distribution of the general population for each country $\mathbb{E}$. We searched for matrices of daily numbers of contacts between each age class $\mathbb{F}$ collected in surveys relevant to all countries. We decided to use a contact matrix measured in Shanghai in 2019 for Hubei[3]. We could not use the alternative contact matrix measured in [14] because it was not available in a useable format, even after asking the authors. The contact matrix projected for China from European POLYMOD data in [15] was judged unsuitable to areas outside of Europe in a subsequent paper [16] and was thus ignored. For European settings we used the contact matrix from the POLYMOD study [17]. Table 2 and Figures S1:S7 summarise the data collected for every area.

Table 2: Details and sources of data collected for the 7 areas.

Area	Period of data collection	Confirmed cases with date of symptom onset (proportion of all confirmed cases)	Reported deaths with date of death (proportion of all reported deaths)	Sources
	$t_1 - t_{\max}$	$\sum_t \mathbb{A}_t$ $(\mathbb{G})$	$\sum_t \mathbb{C}_t$ $(\mathbb{H})$	
Austria	11.03 - 14.04.2020	14,151 (100%)	399 (100%)	[18]
Hubei (China)	01.01 - 11.02.2020	41,092 (100%)	979 (100%)	[19]
Hubei (China) with correction	01.01 - 11.02.2020	41,092 (60.6%)	979 (71.4%)	[19, 20]
Lombardy (Italy)	11.02 - 25.04.2020	57,330 (77.1%)	13,263 (100%)	[21, 22]
Baden-Württemberg (Germany)	03.03 - 16.04.2020	19,445 (62.3%)	802 (100%)	[23]
Bavaria (Germany)	03.03 - 16.04.2020	20,547 (56.2%)	1,049 (100%)	[23]
Spain	02.03 - 16.04.2020	140,249 (78.8%)	19,478 (100%)	[24]
Switzerland	02.03 - 23.04.2020	3,666 (11.0%)	1,302 (100%)	[25]

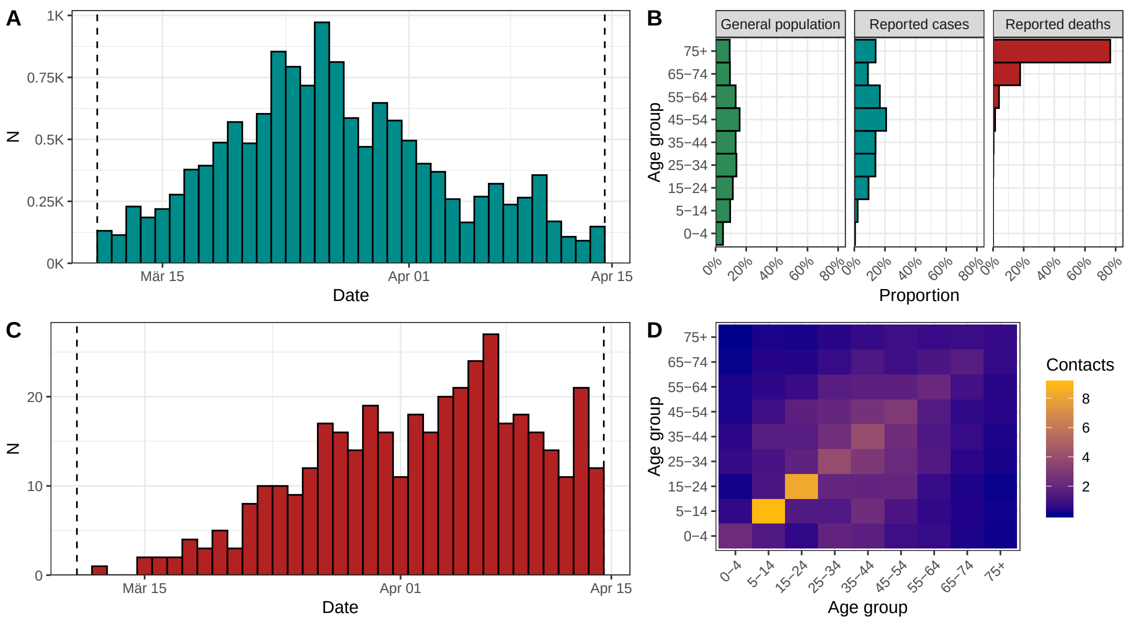

Figure S1: Data used to fit the model in Austria. (A) Confirmed cases of SARS-CoV-2 infection by date of disease onset. (B) Age distribution of the general population, of confirmed cases and of deaths due to SARS-CoV-2. (C) Reported deaths among confirmed cases of SARS-CoV-2 infection. (D) Matrix representing the average number of daily contacts between each age class in Europe (POLYMOD).

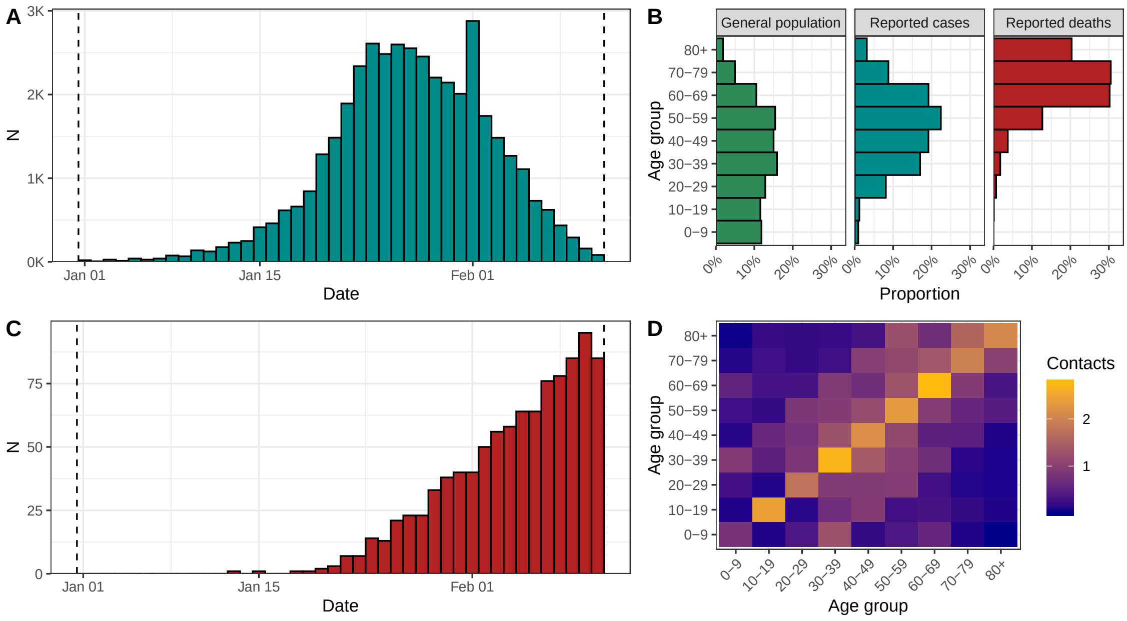

Figure S2: Data used to fit the model in Hubei, China. (A) Confirmed cases of SARS-CoV-2 infection by date of disease onset. (B) Age distribution of the general population, of confirmed cases and of deaths due to SARS-CoV-2. (C) Reported deaths among confirmed cases of SARS-CoV-2 infection. (D) Matrix representing the average number of daily contacts between each age class in Shanghai.

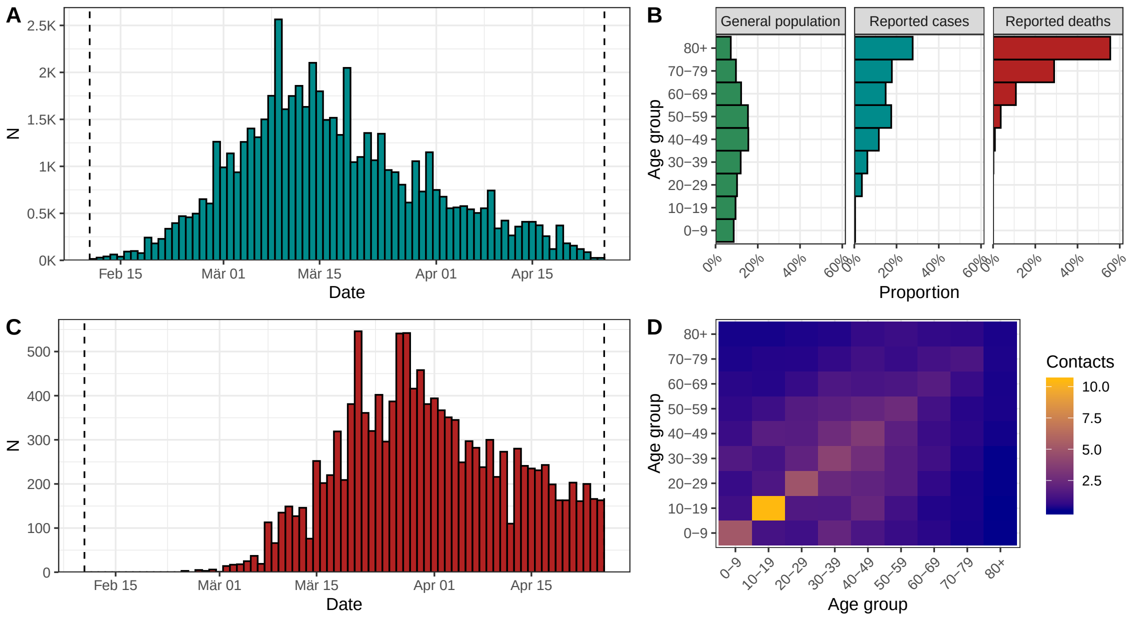

Figure S3: Data used to fit the model in Lombardy, Italy. (A) Confirmed cases of SARS-CoV-2 infection by date of disease onset. (B) Age distribution of the general population, of confirmed cases and of deaths due to SARS-CoV-2. (C) Reported deaths among confirmed cases of SARS-CoV-2 infection. (D) Matrix representing the average number of daily contacts between each age class in Europe (POLYMOD).

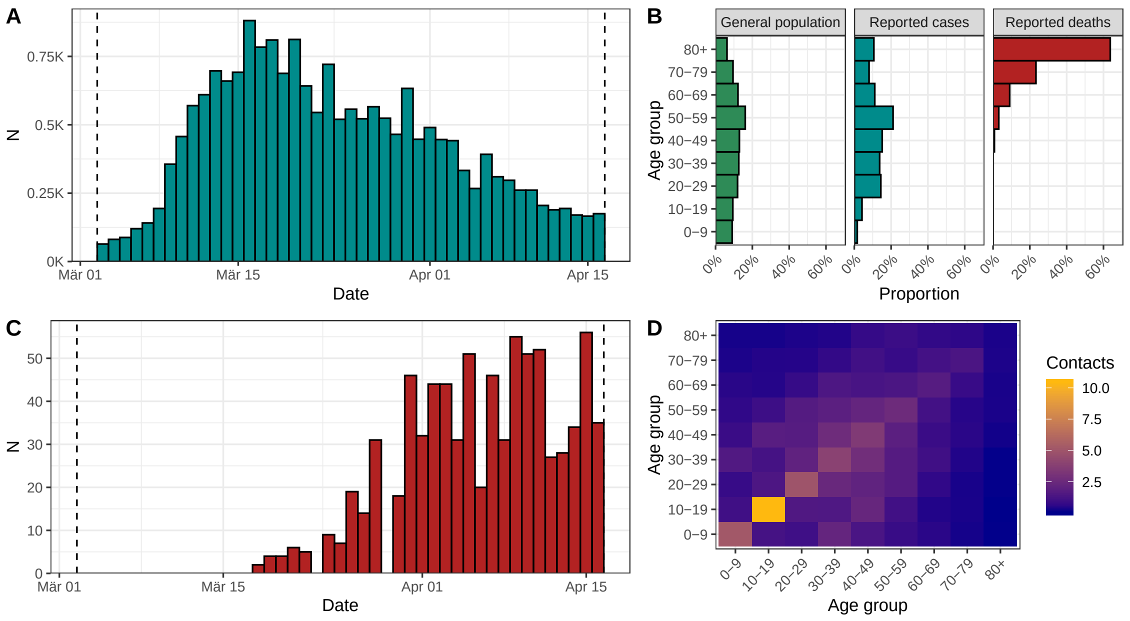

Figure S4: Data used to fit the model in Baden-Württemberg, Germany. (A) Confirmed cases of SARS-CoV-2 infection by date of disease onset. (B) Age distribution of the general population, of confirmed cases and of deaths due to SARS-CoV-2. (C) Reported deaths among confirmed cases of SARS-CoV-2 infection. (D) Matrix representing the average number of daily contacts between each age class in Europe (POLYMOD).

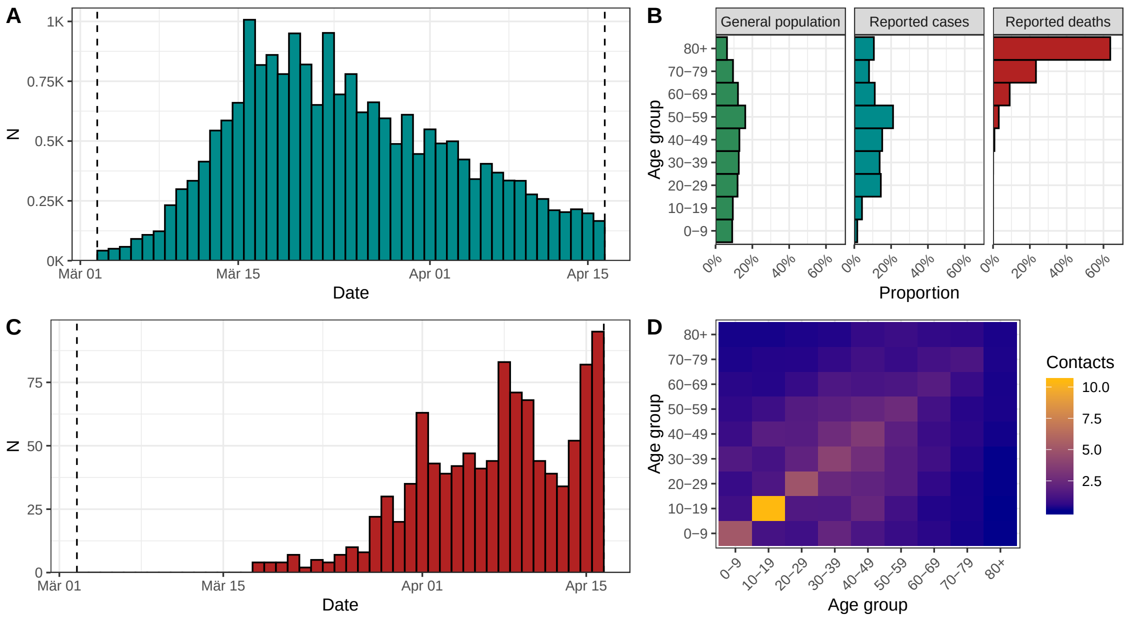

Figure S5: Data used to fit the model in Bavaria, Germany. (A) Confirmed cases of SARS-CoV-2 infection by date of disease onset. (B) Age distribution of the general population, of confirmed cases and of deaths due to SARS-CoV-2. (C) Reported deaths among confirmed cases of SARS-CoV-2 infection. (D) Matrix representing the average number of daily contacts between each age class in Europe (POLYMOD).

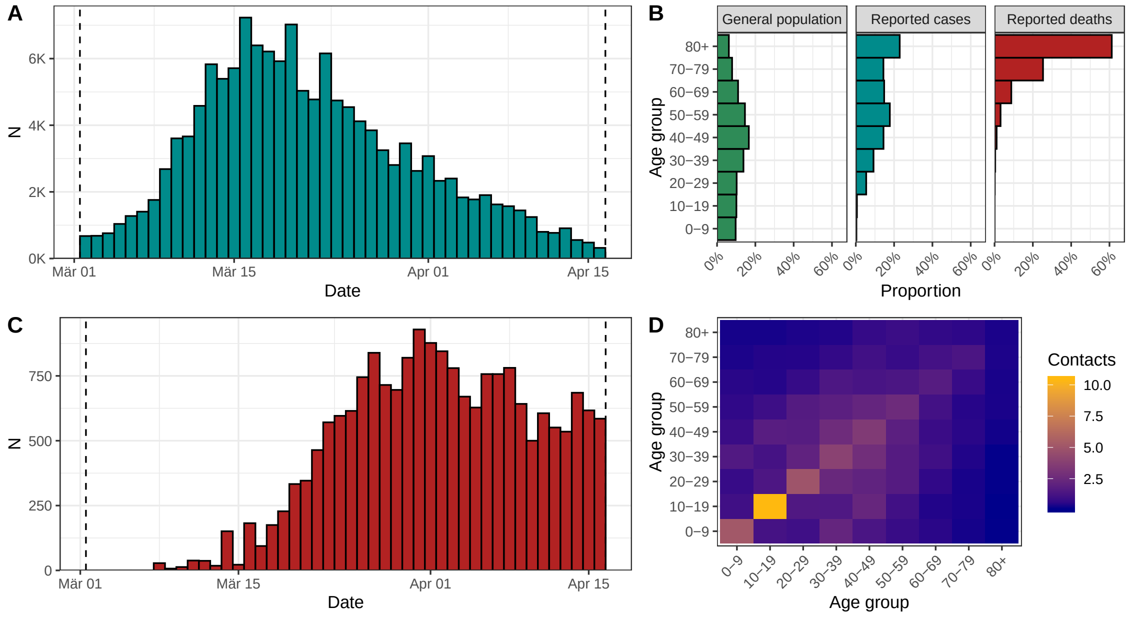

Figure S6: Data used to fit the model in Spain. (A) Confirmed cases of SARS-CoV-2 infection by date of disease onset. (B) Age distribution of the general population, of confirmed cases and of deaths due to SARS-CoV-2. (C) Reported deaths among confirmed cases of SARS-CoV-2 infection. (D) Matrix representing the average number of daily contacts between each age class in Europe (POLYMOD).

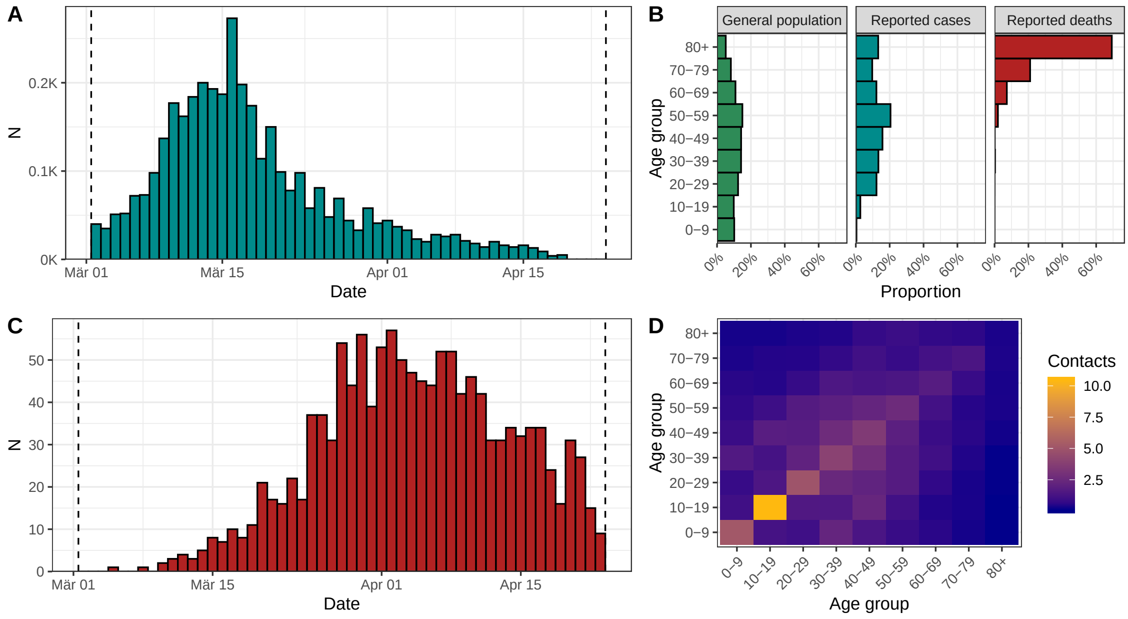

Figure S7: Data used to fit the model in Switzerland. (A) Confirmed cases of SARS-CoV-2 infection by date of disease onset. (B) Age distribution of the general population, of confirmed cases and of deaths due to SARS-CoV-2. (C) Reported deaths among confirmed cases of SARS-CoV-2 infection. (D) Matrix representing the average number of daily contacts between each age class in Europe (POLYMOD).

2 Transmission model

We develop an approach aimed at inferring the symptomatic fatality rate (SFR) and the infection fatality rate (IFR) of SARS-CoV-2 infection from surveillance data. With regards to a specific area, the model relies upon data on confirmed cases of SARS-CoV-2 infection *by date of symptom onset* (which is not always communicated by surveillance authorities), mortality among confirmed cases of SARS-CoV-2 infection by date of death, and the age distribution of both cases and deaths. In a first step, we use an age-stratified susceptible-exposed-infected-removed (SEIR)-type compartmental model to describe the dynamics of transmission of the virus in the population during the first wave of SARS-CoV-2, including the decrease in incidence after the introduction of control measures. In a second step, we estimate the mortality of infected individuals with regards to the probability of dying upon infection and the delay between symptom onset and death. We fit the model to data on the incidence and age distribution of both confirmed cases and deaths. We implement the model in a Bayesian framework using Stan [26]. All code and data are available from https://github.com/jriou/covid_adjusted_cfr/.

2.1 General structure

We use an age-stratified SEIR-type compartmental model using ordinary differential equations (ODEs), with a distinction between presymptomatic, asymptomatic and symptomatic infections. We stratify the population in nine ten-year age groups (0-9 up to 80+ years for all areas except Austria where the 9 age groups are 0-4, 5-14, up to 75+ years). The population of each age group k is divided into six compartments: susceptible (S_k), incubating or exposed (E_k), presymptomatic (P_k), infected with symptoms (I_k), infected without symptoms (A_k), and removed (R_k). Note that the number of individuals in each compartment is scaled by the total population of the area, so that the sum of all compartments is 1. Susceptible individuals may become infected through contacts with infectious individuals (compartments P, I, and A) from any other age group according to an age-specific contact matrix. This contact matrix is a central element of the transmission dynamics as it determines the relative attack rates in different age groups. The system of ODEs is expressed as follows:

$$\frac{dS_k}{dt} = -\lambda_k(t)S_k \tag{1}$$
$$\frac{dE_k}{dt} = \lambda_k(t)S_k - \tau_1 E_k \tag{2}$$
$$\frac{dP_k}{dt} = \tau_1 E_k - \tau_2 P_k \tag{3}$$
$$\frac{dA_k}{dt} = \tau_2(1-\psi)P_k - \mu A_k \tag{4}$$
$$\frac{dI_k}{dt} = \tau_2 \psi P_k - \mu I_k \tag{5}$$
$$\frac{dR_k}{dt} = \mu(A_k + I_k) \tag{6}$$

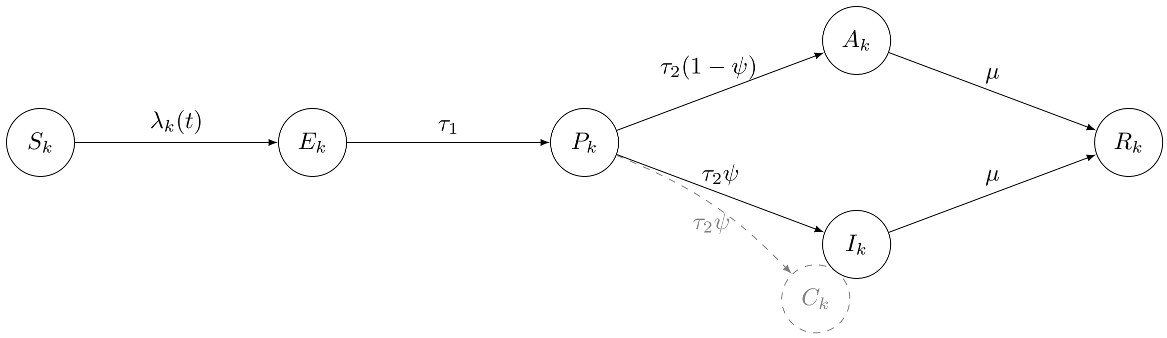

Figure S8: Schematic description of the mode of SARS-CoV-2 transmission. The dashed circle represents the dummy compartment used to record the cumulative incidence of symptomatic cases C_k.

2.2 Force of infection

The time-dependent force of infection $\lambda_k(t)$ for age group $k \in \{1, \ldots, 9\}$ is expressed as:

$$\lambda_k(t) = f(t, \eta, \nu, \xi)\beta \sum_{l=1}^{9} \frac{I_l(t) + \kappa P_l(t) + \kappa A_l(t)}{\mathbb{E}_l} \mathbb{F}_{k,l} \tag{7}$$

It depends on:

- the proportion of infectious individuals in each age class $l \in \{1, \ldots, 9\}$, i.e. compartments I_l, P_l and A_l divided by the total size of the age class $\mathbb{E}_l$;
- the total number of contacts between individuals of age class k and age class l, i.e. the contact matrix $\mathbb{F}$;
- the probability of infection upon contact with an infectious individual β. We consider a unique β for every age class, thus assuming the same susceptibility to infection (this assumption was relaxed in a sensitivity analysis, section 6);
- the transmissibility from presymptomatic and asymptomatic infections reduced by a parameter $\kappa \in [0, 1]$;
- the time-dependent reduction in transmissibility due to control measures $f(t, \eta, \nu, \xi)$.

2.3 Incubation, infectiousness and symptom onset

We propose an approach to model SARS-CoV-2 transmission accounting for asymptomatic and presymptomatic transmission and for published estimates of the incubation period and the generation time. Following infection, we split incubation in two successive periods: first, a period of $1/\tau_1$ days of incubation without transmission, followed by a period of $1/\tau_2$ days of incubation with reduced transmission (that we call presymptomatic transmission). After incubation, a proportion ψ of infected individuals develop symptoms while the remaining remain asymptomatic. After symptom onset, symptomatic individuals may transmit SARS-CoV-2 to their contacts with probability β for a duration $1/\mu$. Transmission from pre- and asymptomatic infections is reduced by κ.

A specificity of the natural history of SARS-CoV-2 infection is the similar average duration of the incubation period and the generation time, both close to 5 days [6, 7, 8, 9, 10, 11]. This implies that a significant part of transmission occurs before symptom onset, and that the infectious period after symptom onset must be relatively short. The contribution of presymptomatic infections to transmission has been estimated to 44-48% [9, 11, 12]. We fix the average duration of the incubation period to 5 days [6, 7, 8, 9, 10], with possible transmission during the last few days of incubation, starting 2.3 days (95% confidence interval, 0.8–3.0) before symptom onset [12]. In consequence, we fix $1/\tau_1$ to 2.7 days and $1/\tau_2$ to 2.3 days.

The duration of the infectious period $1/\mu$ is fixed with regards to the generation time. In simple SEIR models, the generation time corresponds to the sum of the incubation and infectious periods [27, 28]. As in this particular case, transmission can occur during incubation, the generation time must account for the contribution of presymptomatic infections to the overall transmission q. This is expressed as:

$$g = \frac{1}{\tau_1} + q\frac{1}{\tau_2} + (1-q)\left(\frac{1}{\tau_2} + \frac{1}{\mu}\right), \tag{8}$$

from which we derive:

$$\frac{1}{\mu} = \frac{g - \frac{1}{\tau_1} - \frac{1}{\tau_2}}{1-q}. \tag{9}$$

If we fix the generation time to $g = 5.2$ days [11, 12] and the contribution of pre- and asymptomatic infections to transmission to $q = 0.46$ [11, 29, 12], this results in an infectious period of $1/\mu = 0.37$ days. This period is relatively short, but it is expected in order to comply with the duration of the generation time.

2.4 Proportion of symptomatic infections

A meta-analysis estimated the proportion of asymptomatics to 19% $[11\% - 29\%]$ [30]. As some heterogeneity is observed between studies, we defined the proportion of symptomatics ψ as a free parameter, with a prior distribution that reflects this observed uncertainty : $\psi \sim \text{Beta}(58.3, 13.7)$ (Table 3). Implementing this information as a beta distribution allowed the propagation of uncertainty on ψ into the final estimates.

2.5 Contribution of presymptomatic transmission

Once we know the duration of the infectious period $1/\mu$ and the proportion of asymptomatics ψ, we can derive the reduction in transmission κ. Besides μ and ψ, the reduction in transmission κ depends on the contribution of presymptomatic infections to transmission q and the assumed average durations for presymptomatic transmission ($1/\tau_2$). The contribution of presymptomatic infections to transmission can be expressed as:

$$q = \frac{\frac{\kappa}{\tau_2}}{\frac{\kappa}{\tau_2} + (1-\psi)\frac{\kappa}{\mu} + \psi\frac{1}{\mu}}, \tag{10}$$

from which we derive:

$$\kappa = \frac{q\tau_2\psi}{(1-q)\mu - (1-\psi)q\tau_2}. \tag{11}$$

With our choice of values for the other parameters, we obtain a median value for κ of 11% (95%CrI of 10-12).

2.6 Control measures

We include a time-dependent forcing function to account for the reduction in transmissibility following the introduction of control measures. We model the progressive introduction of control measures using the following logistic function:

$$f(t, \eta, \nu, \xi) = \eta + \frac{1-\eta}{1 + \exp(\xi(t - t_c - \nu))} \tag{12}$$

where η is the relative reduction in transmission after control measures are fully implemented, ξ is the slope of the logistic function modelling the implementation of the control measures, and ν is the delay until the control measures are 50% effective (in days after t_c, the date of introduction of control measures).

3 Model simulation

3.1 Initial conditions and simulation settings

The date of simulation start t_0 is set on the day before the date of the first data point t_1 (Table 2). At t_0, most individuals are assumed to be susceptible and are distributed across age groups according to the population age distribution $\mathbb{E}_k$. The number of people in the exposed compartments at t_0 is controlled by a parameter π, and also distributed according to $\mathbb{E}_k$. Other compartments are set to 0.

3.2 Dummy compartments and outputs

In addition to the six compartments per age group, we add one dummy compartment to record the cumulative incidence of symptomatic infections by day of symptom onset, C_k:

$$\frac{dC_k}{dt} = \tau_2\psi P_k \tag{13}$$

Moreover, we need the following outputs in order to fit the model:

- the number of new symptomatic infections per day of symptom onset by age group:

$$\Delta C_{k,t} = C_k(t) - C_k(t-1) \tag{14}$$

- the total number of new infections per day of symptom onset:

$$\Delta D_t = \sum_k^9 \Delta C_{k,t} \tag{15}$$

- the total number of new reported infections per day of symptom onset, introducing the age-specific ascertainment proportion parameter ρ_k and if needed, correcting for the proportion of confirmed cases with a date of symptom onset $\mathbb{G}$ (assuming that the date of symptom onset is missing at random):

$$R_t = \mathbb{G} \sum_k^9 \rho_k \Delta C^I_{k,t} \tag{16}$$

- the age distribution of all reported cases up to $t_{\max}$:

$$D_k^{\text{cases}} = \frac{\rho_k C_k^I(t_{\max})}{\sum_k^9 \rho_k C_k^I(t_{\max})} \tag{17}$$

  where $t_{\max}$ represents the index of the last simulated day (Table 2).

To allow identifiability of the parameters, we remove a degree of freedom by fixing the ascertainment proportion of the highest age group ρ_9 to 100%. This assumes that all infected individuals in the highest age class with symptoms seek medical care, are identified and reported to the authorities. The ascertainment proportions for other groups $\{\rho_1, \ldots, \rho_8\}$ are estimated from data.

3.3 Delayed mortality

Mortality is considered outside of the system of ODEs, using an age-specific mortality parameter ε_k. In accordance to data, we only consider mortality among the true number of infectious individuals with symptoms up to $t_{\max}$. In order to capture the delay between symptom onset and death, we calculated the mortality up 60 days after $t_{\max}$. To do so, we generated a discretized log-normal distribution of time from symptom onset to death $\mathbb{I}$ of length 60 (Figure S9).

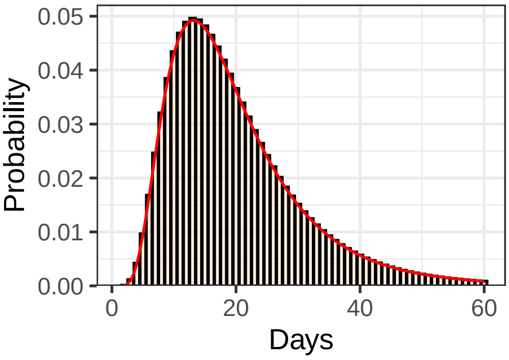

Figure S9: Probability density function of the time from symptom onset to death (red) and corresponding discretization by day between 1 and 60 days (bars).

In order to estimate ε_k, we need the following mortality-related outputs:

- the number of deaths in age group k at time t ($1 \leq t \leq t_{\max} + 60$) among people infected up to $t_{\max}$, to account for the delay between symptom onset and deaths, and if needed corrected for the proportion of deaths with unknown date of death $\mathbb{H}$ (assuming that the date of death is missing at random, this only applies to "Hubei with correction", see Table 2):

$$M_{k,t} = \mathbb{H}\varepsilon_k \sum_{d}^{60} \Delta C_{k,t-d}\mathbb{I}_d \tag{18}$$

- the daily number of deaths summed over age groups, assuming that all deaths are reported:

$$M_t = \sum_{k}^{9} M_{k,t} \tag{19}$$

- the age distribution of all deaths occurring up to $t_{\max}$:

$$D_k^{\text{deaths}} = \frac{\sum_{t=1}^{t_{\max}} M_{k,t}}{\sum_{t=1}^{t_{\max}} M_t} \tag{20}$$

3.4 Inference

The objective of this step is to estimate the following parameters: $\theta = \{\beta, \eta, \xi, \nu, \psi, \pi, \rho_k, \varepsilon_k, \phi_1, \phi_2\}$. We set weakly-informative prior distributions for most of these parameters (Table 3). For the initial proportion of infected, we used a informative prior distribution $\pi \sim \text{Beta}(1, 999)$ corresponding to a 95% central range of 0-0.0037 of the total population. This range is actually quite large and weakly-informative as it corresponds to a time where no or very few cases had been reported.

We jointly fitted the model to four data sets:

1. the number of confirmed cases of SARS-CoV-2 infection by day of symptom onset between t_1 and $t_{\max}$:

$$\Pr(\theta|\mathbb{A}) = \prod_{t=t_1}^{t_{\max}} \text{Neg-Bin}(\mathbb{A}_t|R_t, R_t/\phi_1) \tag{21}$$

2. the age distribution of all confirmed cases up to $t_{\max}$:

$$\Pr(\theta|\mathbb{B}) = \text{Multinomial}(\mathbb{B}|D_k^{\text{cases}}) \tag{22}$$

Symbol	Comment	Support	Prior
β	Probability of transmission upon contact	$[0-1]$	$\text{Beta}(1,1)$
η	Reduction in transmission after control measures	$[0-1]$	$\text{Beta}(1,1)$
ξ	Slope of logistic function for control measures implementation	$[0.5-1.5]$	$\text{Beta}(1,1)+0.5$
ν	Shift of logistic function for control measures implementation	$[0-\infty[$	$\text{Exp}(1)$
ψ	Proportion of symptomatics	[0-1]	$\text{Beta}(58.3,13.7)$
ε_k	Proportion of deaths among symptomatic cases (by age group)	[0-1]	$\text{Beta}(1,1)$
ρ_k	Reporting rate of symptomatic cases (by age group)	[0-1]	$\text{Beta}(1,1)$
π	Initial proportion of exposed (at t_0)	[0-1]	$\text{Beta}(1,999)$
ϕ_1	Overdispersion for cases	$[0-\infty[$	$\text{Exp}(1/100)$
ϕ_2	Overdispersion for deaths	$[0-\infty[$	$\text{Exp}(1/100)$

Table 3: Summary of parameters.

3. the number of deaths among patients with confirmed SARS-CoV-2 infection by day of death:

$$\Pr(\theta|\mathbb{C}) = \prod_{t=t_1}^{t_{\max}} \text{Neg-Bin}(\mathbb{C}_t|M_t, M_t/\phi_2) \tag{23}$$

4. the age distribution of deaths among patients with confirmed SARS-CoV-2 infection that occurred between t_1 and $t_{\max}$:

$$\Pr(\theta|\mathbb{D}) = \text{Multinomial}(\mathbb{D}|D_k^{\text{deaths}}) \tag{24}$$

This leads to the following joint likelihood:

$$\Pr(\theta|\mathbb{A},\mathbb{B},\mathbb{C},\mathbb{D}) = \Pr(\theta|\mathbb{A})\cdot\Pr(\theta|\mathbb{B})\cdot\Pr(\theta|\mathbb{C})\cdot\Pr(\theta|\mathbb{D}) \tag{25}$$

3.5 Estimates of mortality

Table 4 summarizes the different estimates of mortality according to whether we adjust for preferential ascertainment of severe cases and/or right-censoring of deaths.

		Adjusting for preferential ascertainment: No	Adjusting for preferential ascertainment: Yes
Adjusting for right-censoring of deaths	No	$\frac{M_{t_{\max}}}{R_{t_{\max}}}$	$\frac{M_{t_{\max}}}{C^I_{t_{\max}}}$
	Yes	$\frac{M_{t_{\max}+60}}{R_{t_{\max}+60}}$	$\frac{M_{t_{\max}+60}}{C^I_{t_{\max}+60}}$

Table 4: Mortality among symptomatic infections.

The symptomatic fatality rate (SFR) thus corresponds to:

$$SFR = \frac{M_{t_{\max}+60}}{C_{t_{\max}+60}} \tag{26}$$

We also estimate the adjusted mortality in the overall infected (symptomatic or asymptomatic) population:

$$IFR = SFR\cdot\psi \tag{27}$$

3.6 Implementation and diagnostics

We implemented the model in *Stan* 2.18.0 [26], that uses Hamiltonian Monte Carlo sampling. We estimated the posterior distributions by sampling 500 iterations after 500 iterations for warm-up in four independent chains. We assessed convergence visually and by using the Gelman-Rubin convergence diagnostic $\hat{R}$. We also checked for divergent transitions. These diagnostics did not reveal any issue with mixing and convergence in all settings. All posterior predictive checks and trace plots are provided in Figures S10:S15.

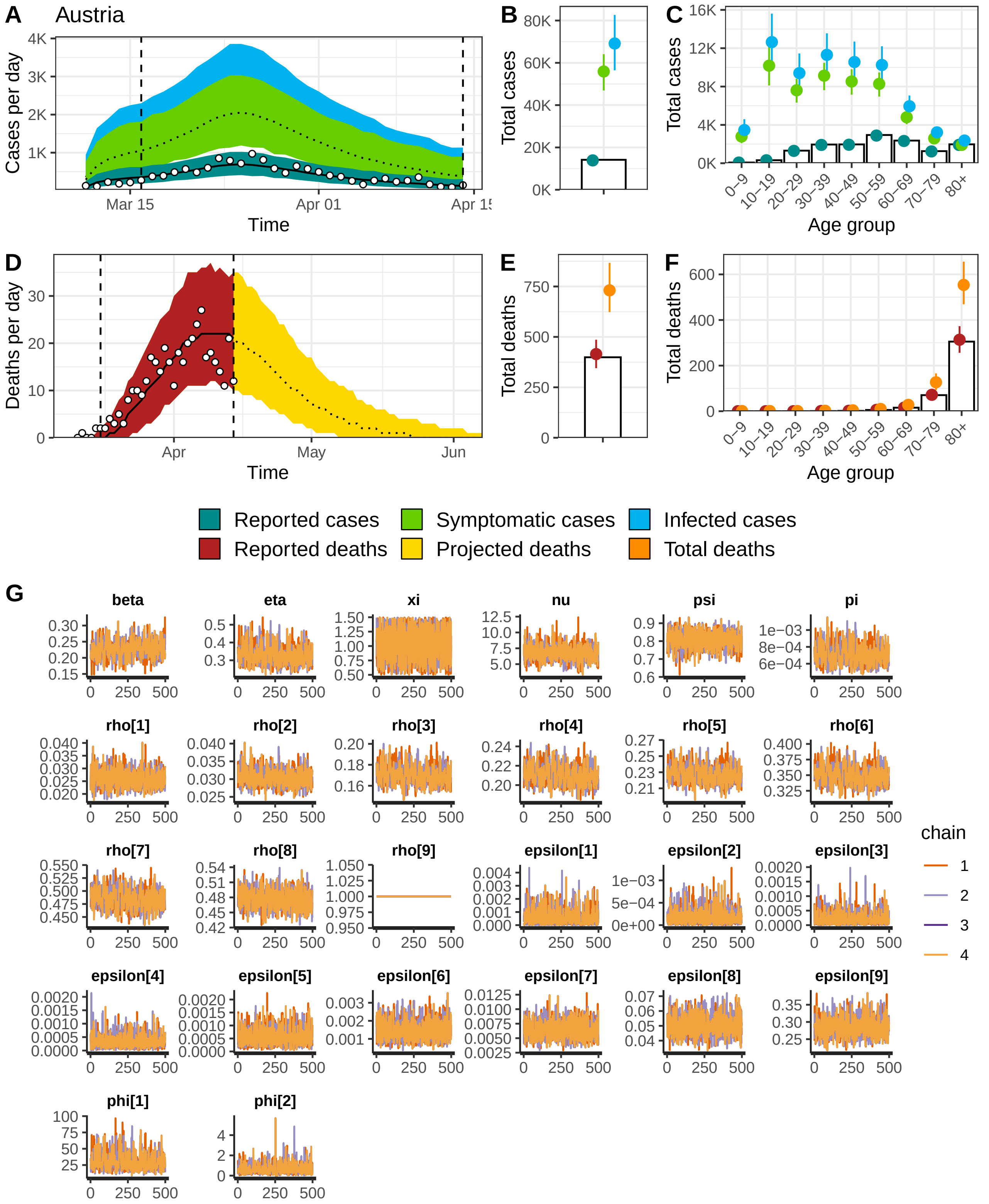

Figure S10: Model fit for Austria of (A) incident cases of SARS-CoV-2 infection by date of symptom onset, (B) total cases, (C) age distribution of cases, (D) incidence of deaths, (E) total number of deaths among individuals infected and (F) age distribution of deaths. White circles and bars represent data. Lines and shaded areas or points and ranges show the posterior median and 95% credible intervals for six types of model output: reported cases, symptomatic cases, overall cases (i.e. symptomatic and asymptomatic cases), reported deaths, projected deaths and overall deaths. Panel (G) shows the trace plot for all parameters (`rho[9]` is fixed to 100%).

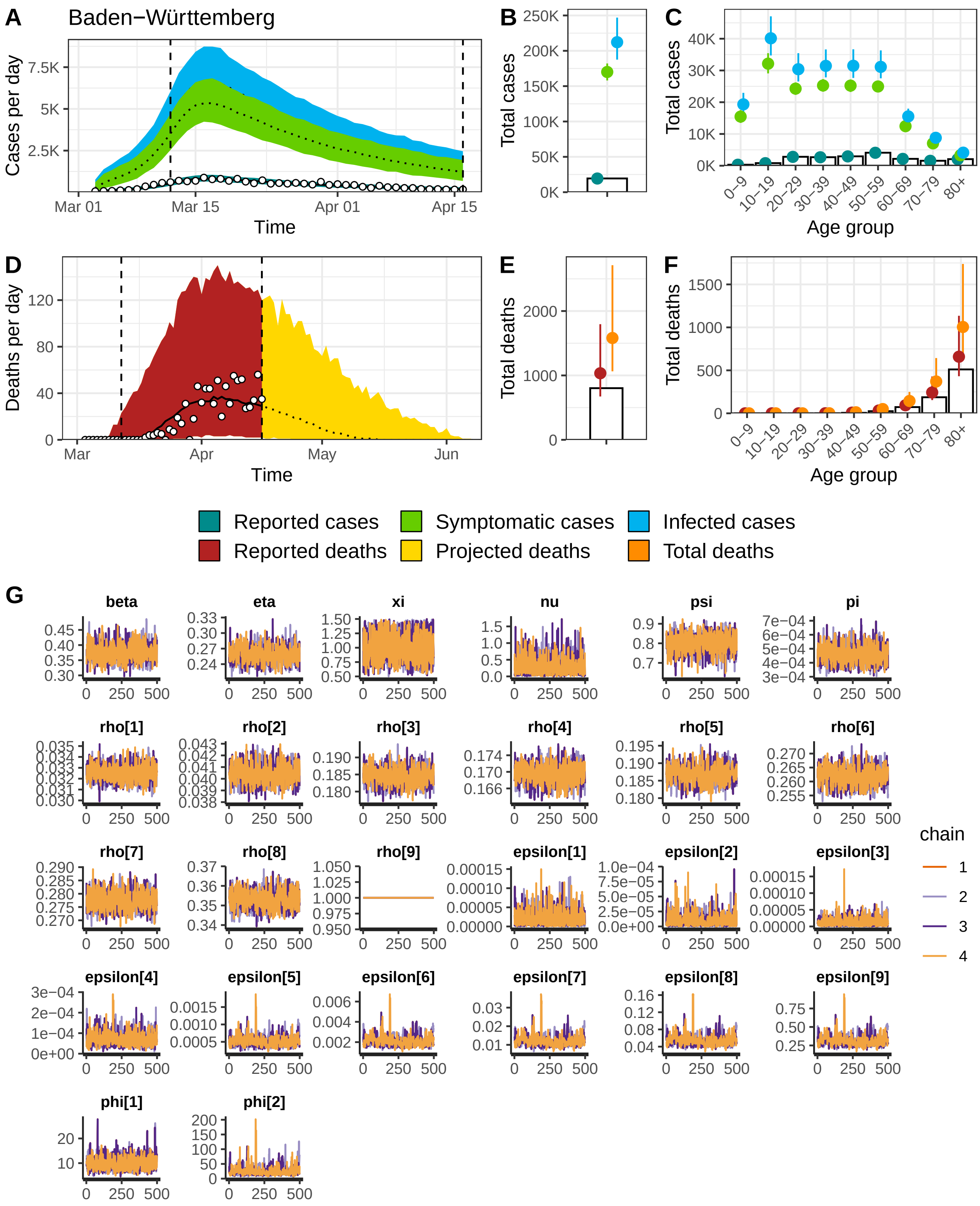

Figure S11: Model fit for Baden-Württemberg of (A) incident cases of SARS-CoV-2 infection by date of symptom onset, (B) total cases, (C) age distribution of cases, (D) incidence of deaths, (E) total number of deaths among individuals infected and (F) age distribution of deaths. White circles and bars represent data. Lines and shaded areas or points and ranges show the posterior median and 95% credible intervals for six types of model output: reported cases, symptomatic cases, overall cases (i.e. symptomatic and asymptomatic cases), reported deaths, projected deaths and overall deaths. Panel (G) shows the trace plot for all parameters (`rho[9]` is fixed to 100%).

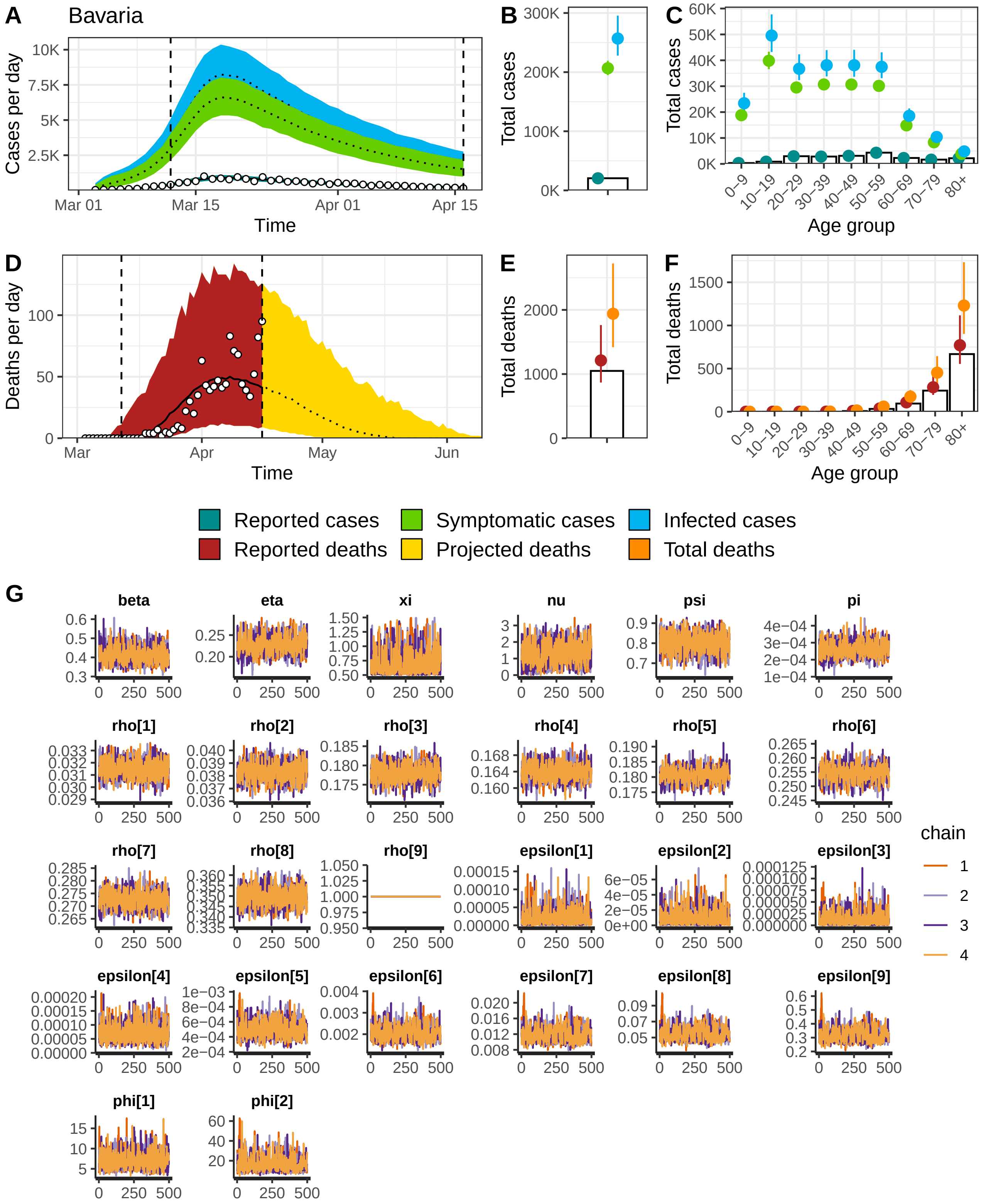

Figure S12: Model fit for Bavaria of (A) incident cases of SARS-CoV-2 infection by date of symptom onset, (B) total cases, (C) age distribution of cases, (D) incidence of deaths, (E) total number of deaths among individuals infected and (F) age distribution of deaths. White circles and bars represent data. Lines and shaded areas or points and ranges show the posterior median and 95% credible intervals for six types of model output: reported cases, symptomatic cases, overall cases (i.e. symptomatic and asymptomatic cases), reported deaths, projected deaths and overall deaths. Panel (G) shows the trace plot for all parameters (`rho[9]` is fixed to 100%).

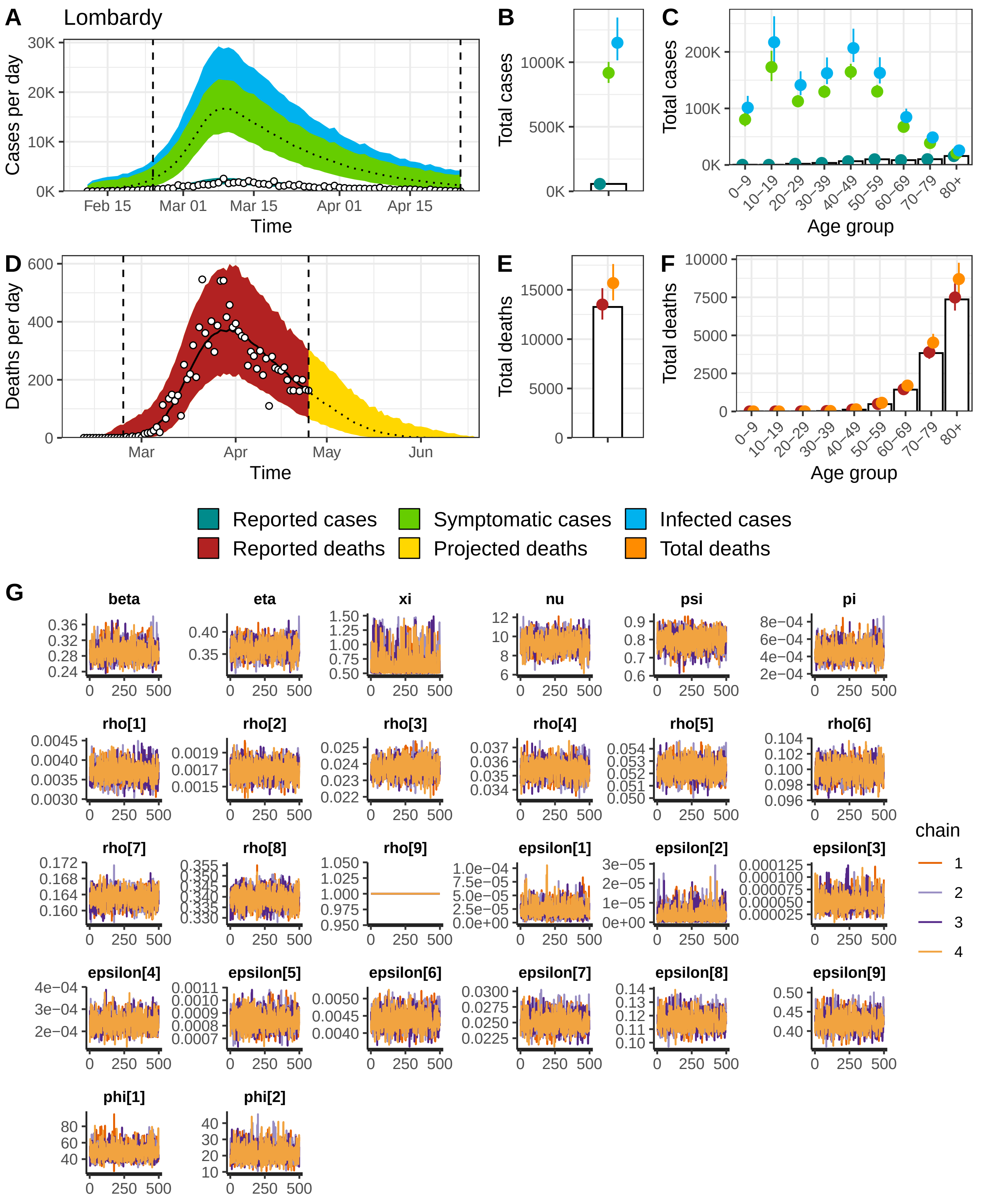

Figure S13: Model fit for Lombardy of (A) incident cases of SARS-CoV-2 infection by date of symptom onset, (B) total cases, (C) age distribution of cases, (D) incidence of deaths, (E) total number of deaths among individuals infected and (F) age distribution of deaths. White circles and bars represent data. Lines and shaded areas or points and ranges show the posterior median and 95% credible intervals for six types of model output: reported cases, symptomatic cases, overall cases (i.e. symptomatic and asymptomatic cases), reported deaths, projected deaths and overall deaths. Panel (G) shows the trace plot for all parameters (`rho[9]` is fixed to 100%).

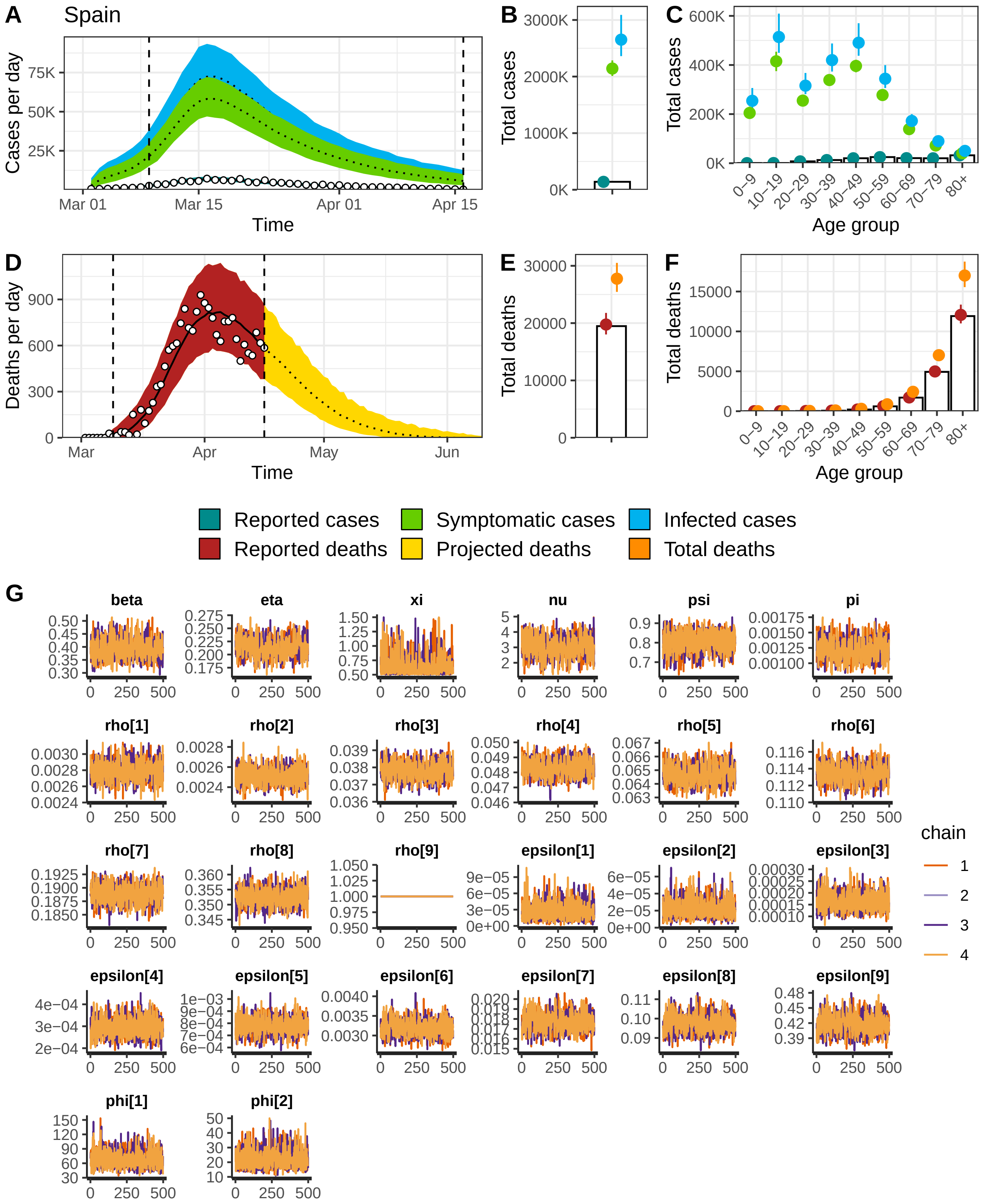

Figure S14: Model fit for Spain of (A) incident cases of SARS-CoV-2 infection by date of symptom onset, (B) total cases, (C) age distribution of cases, (D) incidence of deaths, (E) total number of deaths among individuals infected and (F) age distribution of deaths. White circles and bars represent data. Lines and shaded areas or points and ranges show the posterior median and 95% credible intervals for six types of model output: reported cases, symptomatic cases, overall cases (i.e. symptomatic and asymptomatic cases), reported deaths, projected deaths and overall deaths. Panel (G) shows the trace plot for all parameters (`rho[9]` is fixed to 100%).

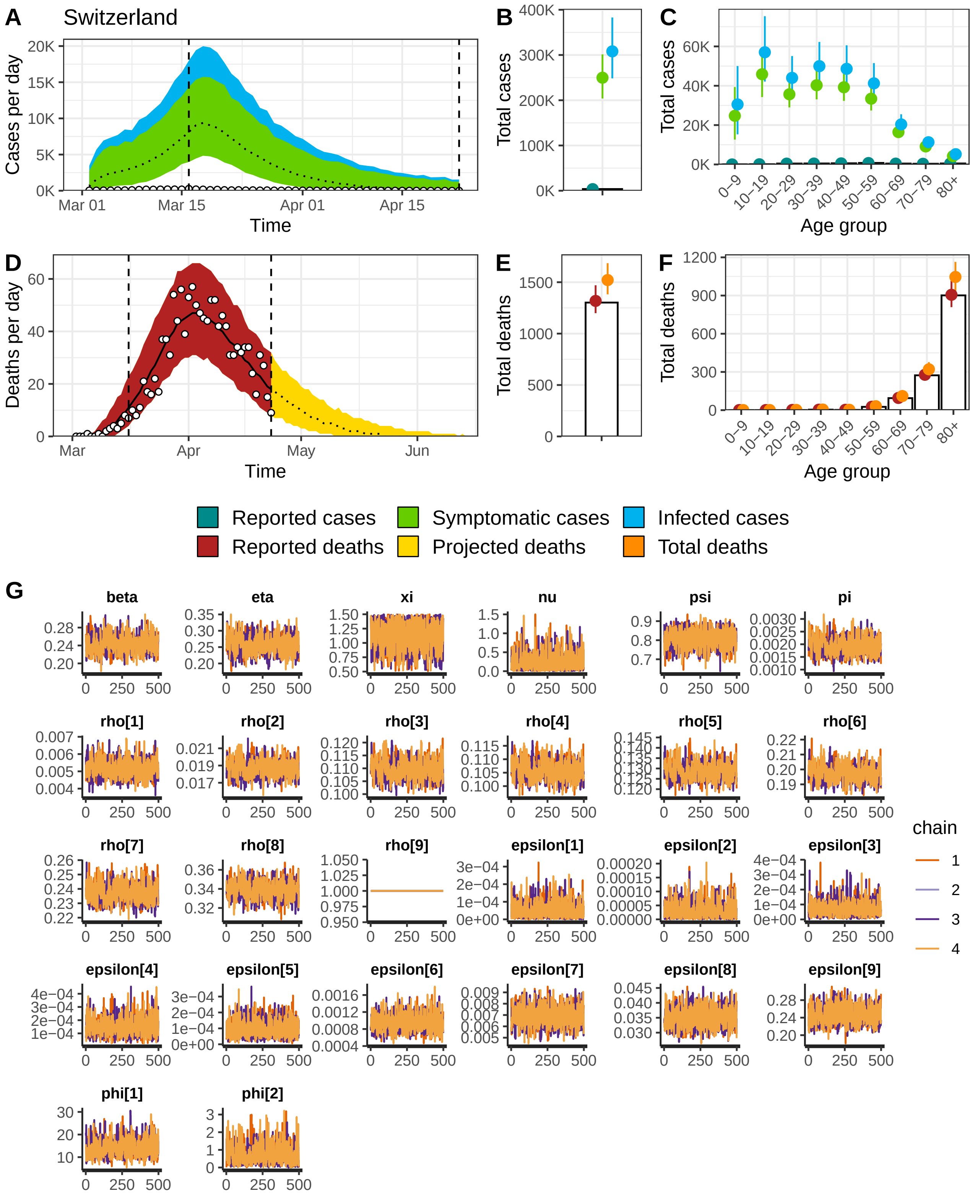

Figure S15: Model fit for Switzerland of (A) incident cases of SARS-CoV-2 infection by date of symptom onset, (B) total cases, (C) age distribution of cases, (D) incidence of deaths, (E) total number of deaths among individuals infected and (F) age distribution of deaths. White circles and bars represent data. Lines and shaded areas or points and ranges show the posterior median and 95% credible intervals for six types of model output: reported cases, symptomatic cases, overall cases (i.e. symptomatic and asymptomatic cases), reported deaths, projected deaths and overall deaths. Panel (G) shows the trace plot for all parameters (`rho[9]` is fixed to 100%).

4 External validation

Some level of external validation of our approach for correcting right-censoring in deaths could be conducted using later reports of deaths in Hubei, China (Figure S16). Visual comparison shows that prediction of deaths align well with later reports (triangles), especially if we average with zero deaths with days with a large number of reported deaths. An important caveat of this comparison is that model prediction only concerns deaths among people infected before 11 February, while later reported deaths may also concern people infected after this date. In consequence, the projections should be lower than the later reports of deaths, because the latter includes deaths among people infected after 11 February (which are very few in Hubei). In any case, the good alignment of predictions and later death reports may serve as an external indication of the adequacy of the model, especially regarding the time between symptom onset and deaths.

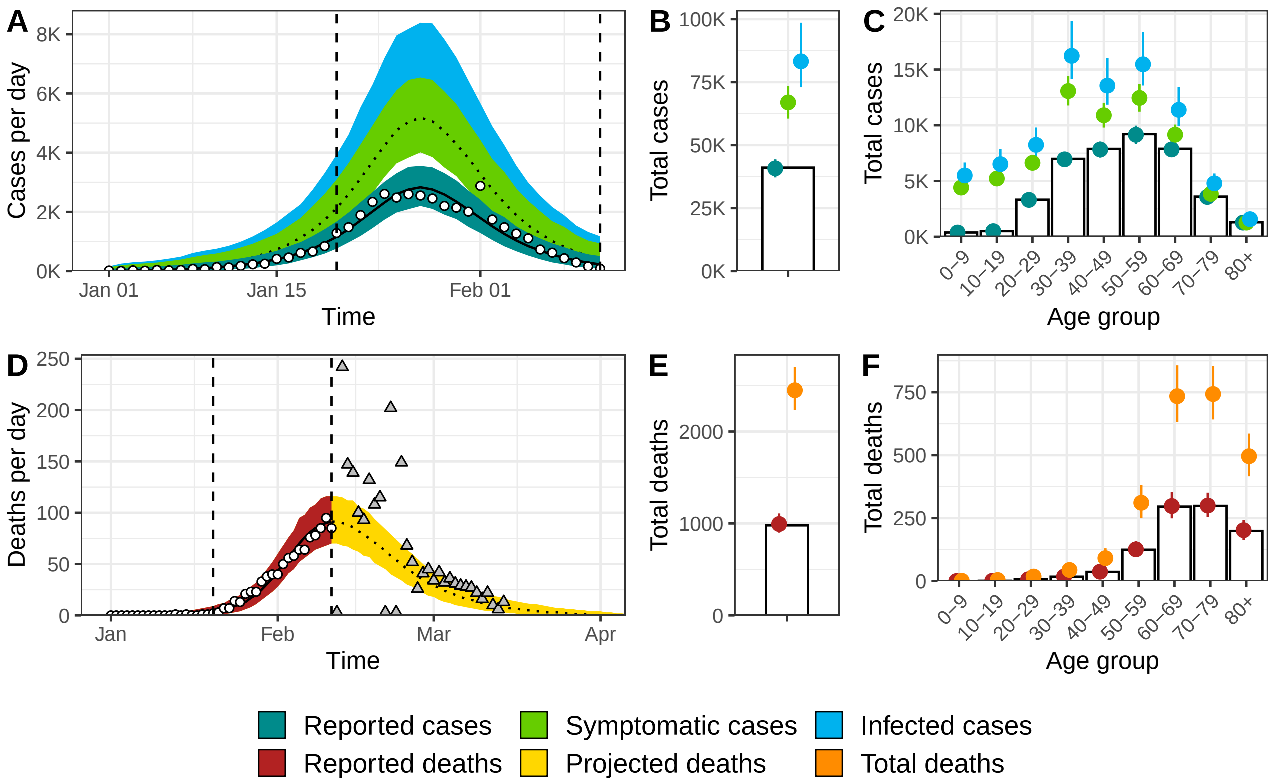

Figure S16: Model fit for Hubei, China of (A) incident cases of SARS-CoV-2 infection by date of symptom onset, (B) total cases, (C) age distribution of cases, (D) incidence of deaths, (E) total deaths and (F) age distribution of deaths. White circles and bars represent data. Lines and shaded areas or points and ranges show the posterior median and 95% credible intervals for five types of model output: reported cases, symptomatic cases, overall cases (i.e. symptomatic and asymptomatic cases), reported deaths until 11 February 2020, and overall deaths including these that will occur after this date.

5 Additional results

5.1 Additional results in Hubei, China

Parameter	Interpretation	Posterior median (95%CrI)
β	Probability of transmission upon contact	0.61 [0.54-0.70]
ψ	Proportion of symptomatic infections	0.81 [0.69-0.89]
π	Initial proportion of infected in the population	0.000006 [0.000004-0.000009]
η	Reduction of transmission due to control measures	0.081 [0.037-0.127]
ν	Delay of implementation of control measures (days)	4.3 [3.2-5.4]
ξ	Slope of implementation of control measures	0.53 [0.50-0.73]
ϕ_1	Overdispersion parameter for cases	38.3 [24.3-64.0]
ϕ_2	Overdispersion parameter for deaths	0.3 [0.0-1.5]

Table 5: Posterior distributions of the general parameters in Hubei, China

Age group	Ascertainment (ρ_k)	SFR (ϵ_k)	IFR ($\epsilon_k \cdot \psi$)
0-9	8.60% [7.77-9.58]	0.038% [0.002-0.198]	0.022% [0.000-0.180]
10-19	9.58% [8.68-10.58]	0.074% [0.010-0.249]	0.058% [0.000-0.223]
20-29	49.86% [46.96-52.87]	0.270% [0.115-0.511]	0.214% [0.071-0.454]
30-39	53.18% [50.20-55.99]	0.338% [0.210-0.514]	0.271% [0.145-0.445]
40-49	71.84% [67.96-75.53]	0.837% [0.605-1.158]	0.676% [0.431-1.006]
50-59	73.45% [69.35-77.24]	2.50% [2.03-3.01]	2.01% [1.52-2.56]
60-69	85.62% [80.88-89.99]	8.04% [6.89-9.30]	6.46% [5.15-7.97]
70-79	93.03% [87.72-98.14]	19.30% [16.73-22.11]	15.45% [12.24-18.77]
80+	100.00% [100.00-100.00]	38.90% [33.05-46.10]	31.43% [24.49-39.33]

Table 6: Posterior distributions of the age-specific parameters in Hubei, China

5.2 Additional results in Austria

Parameter	Interpretation	Posterior median (95%CrI)
β	Probability of transmission upon contact	0.23 [0.18-0.28]
ψ	Proportion of symptomatic infections	0.81 [0.71-0.89]
π	Initial proportion of infected in the population	0.000695 [0.000533-0.000956]
η	Reduction of transmission due to control measures	0.327 [0.252-0.446]
ν	Delay of implementation of control measures (days)	6.7 [4.6-9.3]
ξ	Slope of implementation of control measures	1.06 [0.54-1.49]
ϕ_1	Overdispersion parameter for cases	27.2 [13.8-59.7]
ϕ_2	Overdispersion parameter for deaths	0.5 [0.1-1.8]

Table 7: Posterior distributions of the general parameters in Austria

Age group	Ascertainment (ρ_k)	SFR (ϵ_k)	IFR ($\epsilon_k \cdot \psi$)
0-9	2.57% [2.05-3.26]	0.035% [0.001-0.207]	0.026% [0.000-0.182]
10-19	3.03% [2.65-3.53]	0.011% [0.000-0.062]	0.008% [0.000-0.050]
20-29	16.99% [15.54-18.88]	0.014% [0.001-0.073]	0.010% [0.000-0.067]
30-39	21.02% [19.40-23.12]	0.027% [0.004-0.091]	0.018% [0.000-0.083]
40-49	22.68% [20.82-25.04]	0.048% [0.012-0.135]	0.035% [0.000-0.110]
50-59	34.98% [32.43-38.09]	0.14% [0.06-0.26]	0.10% [0.03-0.22]
60-69	48.50% [45.11-51.99]	0.63% [0.39-0.95]	0.47% [0.24-0.82]
70-79	47.70% [44.16-51.17]	5.00% [3.87-6.44]	3.96% [2.76-5.44]
80+	100.00% [100.00-100.00]	28.43% [23.81-34.65]	23.27% [18.04-30.29]

Table 8: Posterior distributions of the age-specific parameters in Austria

5.3 Additional results in Baden-Württemberg, Germany

Parameter	Interpretation	Posterior median (95%CrI)
β	Probability of transmission upon contact	0.37 [0.32-0.44]
ψ	Proportion of symptomatic infections	0.80 [0.70-0.88]
π	Initial proportion of infected in the population	0.000459 [0.000346-0.000600]
η	Reduction of transmission due to control measures	0.258 [0.231-0.291]
ν	Delay of implementation of control measures (days)	0.2 [0.0-1.0]
ξ	Slope of implementation of control measures	0.96 [0.57-1.46]
ϕ_1	Overdispersion parameter for cases	9.8 [5.9-16.0]
ϕ_2	Overdispersion parameter for deaths	22.3 [9.8-66.2]

Table 9: Posterior distributions of the general parameters in Baden-Württemberg, Germany

Age group	Ascertainment (ρ_k)	SFR (ϵ_k)	IFR ($\epsilon_k \cdot \psi$)
0-9	3.25% [3.12-3.40]	0.001% [0.000-0.007]	0.000% [0.000-0.010]
10-19	4.04% [3.89-4.21]	0.001% [0.000-0.004]	0.000% [0.000-0.005]
20-29	18.43% [17.96-18.97]	0.001% [0.000-0.004]	0.000% [0.000-0.007]
30-39	16.95% [16.49-17.40]	0.007% [0.002-0.015]	0.003% [0.000-0.018]
40-49	18.71% [18.20-19.20]	0.050% [0.031-0.087]	0.038% [0.015-0.078]
50-59	26.21% [25.57-26.85]	0.20% [0.14-0.34]	0.16% [0.09-0.28]
60-69	27.78% [27.15-28.46]	1.16% [0.84-1.92]	0.93% [0.56-1.61]
70-79	35.33% [34.48-36.16]	5.26% [3.87-8.51]	4.20% [2.67-7.23]
80+	100.00% [100.00-100.00]	30.25% [22.38-49.02]	24.40% [15.83-41.99]

Table 10: Posterior distributions of the age-specific parameters in Baden-Württemberg, Germany

5.4 Additional results in Bavaria, Germany

Parameter	Interpretation	Posterior median (95%CrI)
β	Probability of transmission upon contact	0.42 [0.34-0.51]
ψ	Proportion of symptomatic infections	0.80 [0.71-0.89]
π	Initial proportion of infected in the population	0.000258 [0.000182-0.000351]
η	Reduction of transmission due to control measures	0.225 [0.190-0.268]
ν	Delay of implementation of control measures (days)	1.3 [0.1-2.7]
ξ	Slope of implementation of control measures	0.62 [0.50-1.30]
ϕ_1	Overdispersion parameter for cases	7.0 [4.1-11.8]
ϕ_2	Overdispersion parameter for deaths	14.9 [7.8-34.9]

Table 11: Posterior distributions of the general parameters in Bavaria, Germany

Age group	Ascertainment (ρ_k)	SFR (ϵ_k)	IFR ($\epsilon_k \cdot \psi$)
0-9	3.15% [3.02-3.28]	0.001% [0.000-0.007]	0.000% [0.000-0.009]
10-19	3.83% [3.70-3.96]	0.001% [0.000-0.004]	0.000% [0.000-0.004]
20-29	17.81% [17.38-18.23]	0.001% [0.000-0.005]	0.000% [0.000-0.006]
30-39	16.40% [16.00-16.79]	0.006% [0.002-0.014]	0.005% [0.000-0.016]
40-49	18.06% [17.59-18.49]	0.049% [0.033-0.073]	0.038% [0.017-0.070]
50-59	25.47% [24.90-25.99]	0.21% [0.15-0.29]	0.16% [0.10-0.26]
60-69	27.28% [26.65-27.88]	1.19% [0.92-1.63]	0.94% [0.64-1.39]
70-79	34.96% [34.16-35.78]	5.41% [4.27-7.38]	4.34% [3.04-6.37]
80+	100.00% [100.00-100.00]	31.72% [25.04-43.26]	25.54% [18.39-37.40]

Table 12: Posterior distributions of the age-specific parameters in Bavaria, Germany

5.5 Additional results in Lombardy, Italy

Parameter	Interpretation	Posterior median (95%CrI)
β	Probability of transmission upon contact	0.29 [0.25-0.34]
ψ	Proportion of symptomatic infections	0.80 [0.69-0.88]
π	Initial proportion of infected in the population	0.000450 [0.000295-0.000665]
η	Reduction of transmission due to control measures	0.363 [0.329-0.398]
ν	Delay of implementation of control measures (days)	9.3 [7.6-11.0]
ξ	Slope of implementation of control measures	0.57 [0.50-1.28]
ϕ_1	Overdispersion parameter for cases	48.1 [35.2-68.5]
ϕ_2	Overdispersion parameter for deaths	21.0 [13.6-32.9]

Table 13: Posterior distributions of the general parameters in Lombardy, Italy

Age group	Ascertainment (ρ_k)	SFR (ϵ_k)	IFR ($\epsilon_k \cdot \psi$)
0-9	0.37% [0.33-0.42]	0.002% [0.000-0.005]	0.002% [0.000-0.006]
10-19	0.17% [0.15-0.19]	0.000% [0.000-0.001]	0.000% [0.000-0.002]
20-29	2.37% [2.28-2.47]	0.005% [0.002-0.009]	0.004% [0.001-0.009]
30-39	3.54% [3.42-3.66]	0.024% [0.017-0.032]	0.019% [0.011-0.030]
40-49	5.23% [5.10-5.38]	0.084% [0.071-0.098]	0.067% [0.050-0.087]
50-59	9.99% [9.77-10.21]	0.44% [0.39-0.50]	0.35% [0.28-0.42]
60-69	16.32% [15.94-16.69]	2.51% [2.26-2.81]	2.00% [1.63-2.40]
70-79	33.93% [33.13-34.72]	11.65% [10.52-12.97]	9.25% [7.60-11.07]
80+	100.00% [100.00-100.00]	42.52% [38.35-47.10]	33.88% [27.76-40.11]

Table 14: Posterior distributions of the age-specific parameters in Lombardy, Italy

5.6 Additional results in Spain

Parameter	Interpretation	Posterior median (95%CrI)
β	Probability of transmission upon contact	0.39 [0.33-0.48]
ψ	Proportion of symptomatic infections	0.80 [0.70-0.89]
π	Initial proportion of infected in the population	0.001213 [0.000930-0.001552]
η	Reduction of transmission due to control measures	0.214 [0.181-0.249]
ν	Delay of implementation of control measures (days)	3.0 [1.7-4.3]
ξ	Slope of implementation of control measures	0.59 [0.50-1.14]
ϕ_1	Overdispersion parameter for cases	65.5 [42.1-107.0]
ϕ_2	Overdispersion parameter for deaths	21.6 [13.8-36.8]

Table 15: Posterior distributions of the general parameters in Spain

Age group	Ascertainment (ρ_k)	SFR (ϵ_k)	IFR ($\epsilon_k \cdot \psi$)
0-9	0.28% [0.26-0.30]	0.002% [0.001-0.006]	0.002% [0.000-0.006]
10-19	0.25% [0.24-0.27]	0.002% [0.001-0.005]	0.002% [0.000-0.004]
20-29	3.78% [3.69-3.88]	0.016% [0.010-0.024]	0.013% [0.007-0.021]
30-39	4.83% [4.73-4.94]	0.029% [0.022-0.038]	0.023% [0.016-0.032]
40-49	6.47% [6.35-6.61]	0.078% [0.065-0.091]	0.061% [0.047-0.078]
50-59	11.35% [11.14-11.56]	0.32% [0.28-0.36]	0.25% [0.20-0.30]
60-69	18.90% [18.58-19.21]	1.77% [1.60-1.95]	1.41% [1.17-1.66]
70-79	35.25% [34.72-35.81]	9.71% [8.93-10.64]	7.82% [6.51-9.14]
80+	100.00% [100.00-100.00]	41.66% [38.40-45.62]	33.74% [28.20-39.45]

Table 16: Posterior distributions of the age-specific parameters in Spain

5.7 Additional results in Switzerland

Parameter	Interpretation	Posterior median (95%CrI)
β	Probability of transmission upon contact	0.24 [0.21-0.28]
ψ	Proportion of symptomatic infections	0.81 [0.71-0.89]
π	Initial proportion of infected in the population	0.001895 [0.001338-0.002581]
η	Reduction of transmission due to control measures	0.254 [0.205-0.310]
ν	Delay of implementation of control measures (days)	0.2 [0.0-0.8]
ξ	Slope of implementation of control measures	1.19 [0.59-1.48]
ϕ_1	Overdispersion parameter for cases	13.7 [8.5-23.0]
ϕ_2	Overdispersion parameter for deaths	0.6 [0.0-2.0]

Table 17: Posterior distributions of the general parameters in Switzerland

Age group	Ascertainment (ρ_k)	SFR (ϵ_k)	IFR ($\epsilon_k \cdot \psi$)
0-9	0.52% [0.42-0.62]	0.003% [0.000-0.016]	0.003% [0.000-0.018]
10-19	1.86% [1.69-2.06]	0.002% [0.000-0.011]	0.002% [0.000-0.010]
20-29	10.93% [10.28-11.69]	0.005% [0.001-0.019]	0.004% [0.000-0.018]
30-39	10.57% [9.99-11.29]	0.014% [0.004-0.030]	0.010% [0.002-0.029]
40-49	12.83% [12.13-13.74]	0.008% [0.002-0.022]	0.006% [0.000-0.021]
50-59	19.71% [18.72-20.88]	0.09% [0.06-0.13]	0.07% [0.04-0.13]
60-69	23.73% [22.56-25.04]	0.68% [0.52-0.86]	0.54% [0.37-0.78]
70-79	33.83% [32.25-35.67]	3.48% [2.92-4.15]	2.82% [2.16-3.74]
80+	100.00% [100.00-100.00]	24.52% [21.05-28.64]	20.03% [15.61-25.75]

Table 18: Posterior distributions of the age-specific parameters in Switzerland

6 Sensitivity analyses

6.1 Correction of cases and deaths in Hubei, China

From 12 February 2020, Chinese authorities changed their criteria for counting diagnoses of the virus, leading to a jump of more 26,667 new cases to the 41,092 confirmed cases up to February 11. Such a jump was also observed in reported deaths on 16 April, when Wuhan city revised its death toll by adding 1290 deaths to the 3,222 reported up to this date. To investigate the impact of this later correction of both cases and deaths in Hubei province, we ran a sensitivity analysis with corrected numbers of cases and deaths.

In both cases, the date of symptom onset (or the date of death) was not released. To account for these additional cases, we thus assumed that the date of symptom onset (or the date of death) was missing at random, and multiplied the model output by a correction factor $\mathbb{G} = 41,092/67,759 = 60.6\%$ for the cases and $\mathbb{H} = 3,222/4,512 = 71.4\%$ for the deaths (Equations 16 and 18).

The correction of the number of reported cases and deaths in Hubei by the local authorities did not influence the ascertainment proportion (Figure S17A) but led to a proportional decrease of the SFR and IFR estimates by 15% as expected from the correction applied ($0.606/0.714 = 0.85$, Figure S17 and Table 2).

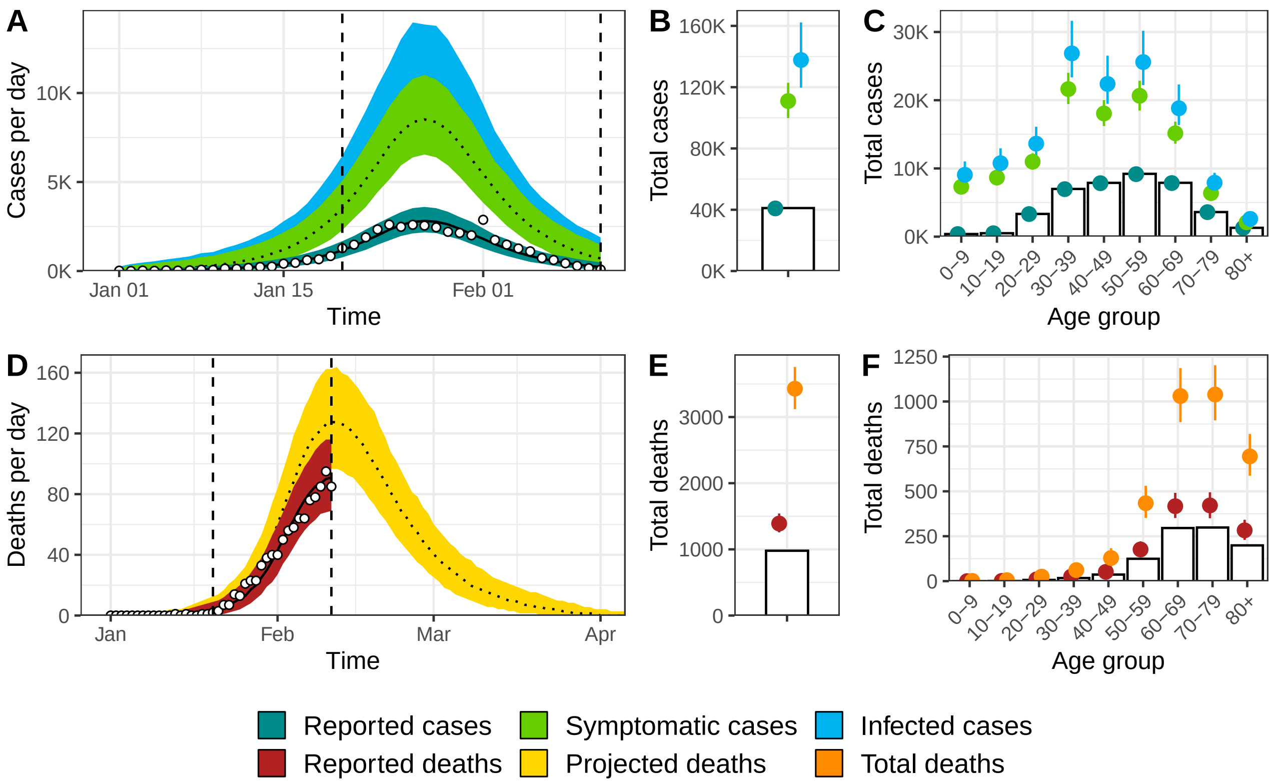

Figure S17: Model fit for Hubei, China **after the correction of cases and deaths**: (A) incident cases of SARS-CoV-2 infection by date of symptom onset, (B) total cases, (C) age distribution of cases, (D) incidence of deaths, (E) total deaths and (F) age distribution of deaths. White circles and bars represent data. Lines and shaded areas or points and ranges show the posterior median and 95% credible intervals for five types of model output: reported cases, symptomatic cases, overall cases (i.e. symptomatic and asymptomatic cases), reported deaths until 11 February 2020, and overall deaths including these that will occur after this date.

6.2 Lower susceptibility in 0-19 year olds

We tested the impact of assuming lower susceptibility among individuals aged 0-19 year old. We reduced the probability of infection upon contact for the two age groups 0-9 and 10-19 years old by 50%. We obtained higher estimates of the ascertainment proportion in the 0-9 age group (18.6% vs 8.6% in main analysis) and in the 10-19 age group (24.1% vs 9.6% in main analysis). As a consequence, the estimated SFR for both classes increased proportionally (0.08% vs 0.04% in 0-9 year olds and 0.19% vs 0.07% in 10-19 year olds), although it remained at very low level. The overall SFR and IFR estimates also slightly increased, from 3.7% to 4.1% for SFR and from 2.9% to 3.3% for SFR.

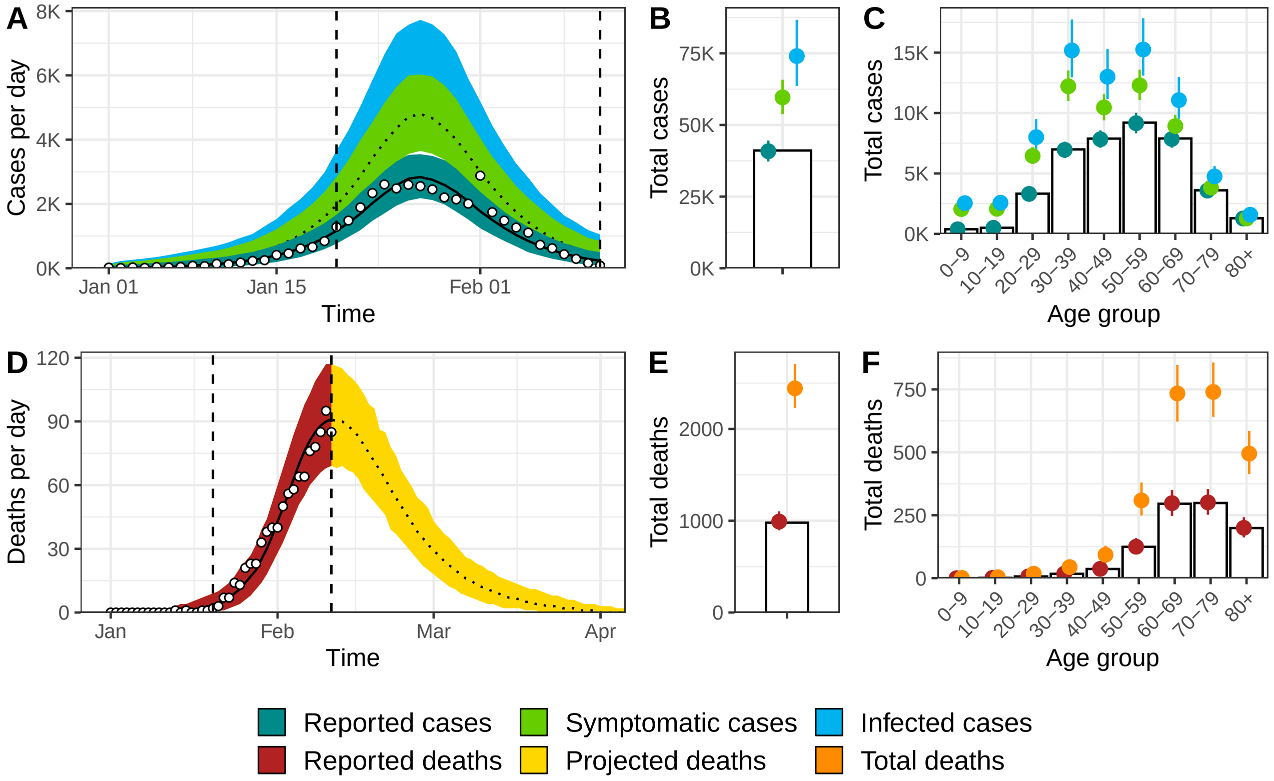

Figure S18: Model fit for Hubei, China **when susceptibility is reduced by 50% in individuals aged 0-19**: (A) incident cases of SARS-CoV-2 infection by date of symptom onset, (B) total cases, (C) age distribution of cases, (D) incidence of deaths, (E) total deaths and (F) age distribution of deaths. White circles and bars represent data. Lines and shaded areas or points and ranges show the posterior median and 95% credible intervals for five types of model output: reported cases, symptomatic cases, overall cases (i.e. symptomatic and asymptomatic cases), reported deaths until 11 February 2020, and overall deaths including these that will occur after this date.

6.3 Ascertainment in the highest age class

In the main analysis, our results depended on the assumption that older individuals that have more severe symptoms are very likely to be identified (ascertainment proportion of 100% for people aged 80 and more with symptoms). In the absence of an outside reference point, the reporting rate cannot be estimated from surveillance data only, and an assumption has to be made. In this sensitivity analysis, we make the assumption that between 10% and 90% of all people aged 80 years and more that were infected with SARS-CoV-2 and had symptoms were identified and reported as cases. This modification leads to a linear scaling of the results as shown in Figure 3C of the main paper.

Table 19: Posterior estimates (median and 95%CrI) of mortality according to the fixed ascertainment rate of infected individuals aged 80 and more with symptoms.

Ascertainment of 80+ with symptoms (ρ_9)	Total cases	Total deaths	CFR	SFR	IFR
10%	832,000 (726,000-995,000)	2450 (2220-2700)	2.4% (2.1-2.8)	0.4% (0.3-0.4)	0.3% (0.2-0.3)
20%	416,000 (363,000-490,000)	2440 (2230-2690)	2.4% (2.1-2.8)	0.7% (0.6-0.8)	0.6% (0.5-0.7)
30%	277,000 (242,000-326,000)	2440 (2240-2690)	2.4% (2.1-2.8)	1.1% (1.0-1.3)	0.9% (0.7-1.0)
40%	208,000 (179,000-244,000)	2440 (2220-2700)	2.4% (2.1-2.8)	1.5% (1.3-1.7)	1.2% (1.0-1.4)
50%	167,000 (144,000-198,000)	2450 (2220-2710)	2.4% (2.1-2.8)	1.8% (1.6-2.1)	1.5% (1.2-1.7)
60%	138,000 (119,000-162,000)	2440 (2220-2680)	2.4% (2.1-2.8)	2.2% (1.9-2.5)	1.8% (1.5-2.1)
70%	119,000 (104,000-140,000)	2450 (2230-2700)	2.4% (2.1-2.8)	2.6% (2.2-2.9)	2.1% (1.7-2.4)
80%	104,000 (90,400-123,000)	2450 (2230-2700)	2.4% (2.1-2.8)	2.9% (2.5-3.4)	2.3% (2.0-2.8)
90%	92,800 (80,200-109,000)	2440 (2210-2700)	2.4% (2.1-2.8)	3.3% (2.9-3.8)	2.6% (2.2-3.1)
Baseline (100%)	83,300 (73,000-98,600)	2450 (2230-2700)	2.4% (2.1-2.8)	3.7% (3.2-4.2)	2.9% (2.4-3.5)

6.4 Earlier date of analysis

In order to better understand the impact of the stage of the epidemic in the estimates of mortality, we refitted the model on data from Hubei truncated to 6 different dates between 12 January and 6 February. The estimates of CFR, SFR and IFR at these dates is shown in Figure 3C of the main paper. Here we show additional results (Table 20) and model fits (Figures S19:S24). The results suggest that the model is not able to properly estimate the total size of the epidemic until after the peak.

Table 20: Posterior estimates (median and 95%CrI) of mortality according to the date of analysis.

Date of analysis	Total cases	Total deaths	CFR	SFR	IFR
January 12	1,620 (1,150-2,240)	102 (1-443)	0.0%	7.8% (0.1-34.0)	6.3% (0.1-27.6)
January 17	5,660 (4,770-6,850)	420 (194-758)	0.2%	9.2% (4.2-16.8)	7.5% (3.2-13.3)
January 22	18,300 (16,100-21,600)	1260 (760-1710)	0.5%	8.5% (5.1-11.4)	6.8% (4.1-9.3)
January 27	43,800 (38,900-50,600)	2190 (1580-3400)	0.6%	6.2% (4.4-9.6)	5.0% (3.4-7.8)
February 1	67,400 (59,300-78,700)	2470 (2100-2940)	0.9%	4.5% (3.8-5.5)	3.7% (2.9-4.6)
February 6	80,200 (70,200-94,000)	2470 (2170-2840)	1.5%	3.8% (3.3-4.5)	3.1% (2.5-3.7)
Baseline (February 11)	83,300 (73,000-98,600)	2450 (2230-2700)	2.4%	3.7% (3.2-4.2)	2.9% (2.4-3.5)

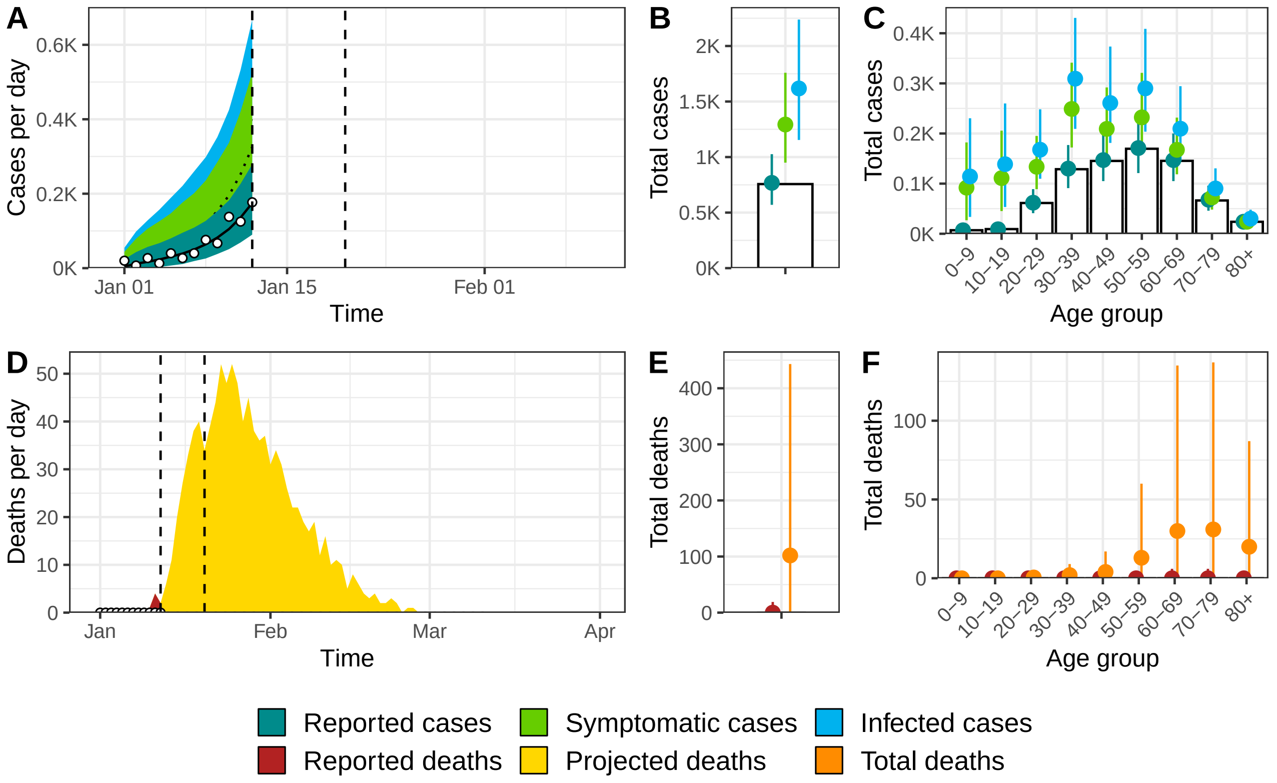

Figure S19: Model fit for Hubei, China **when the analysis is conducted until 12 January**: (A) incident cases of SARS-CoV-2 infection by date of symptom onset, (B) total cases, (C) age distribution of cases, (D) incidence of deaths, (E) total deaths and (F) age distribution of deaths. White circles and bars represent data. Lines and shaded areas or points and ranges show the posterior median and 95% credible intervals for five types of model output: reported cases, symptomatic cases, overall cases (i.e. symptomatic and asymptomatic cases), reported deaths until 17 January 2020, and overall deaths including these that will occur after this date.

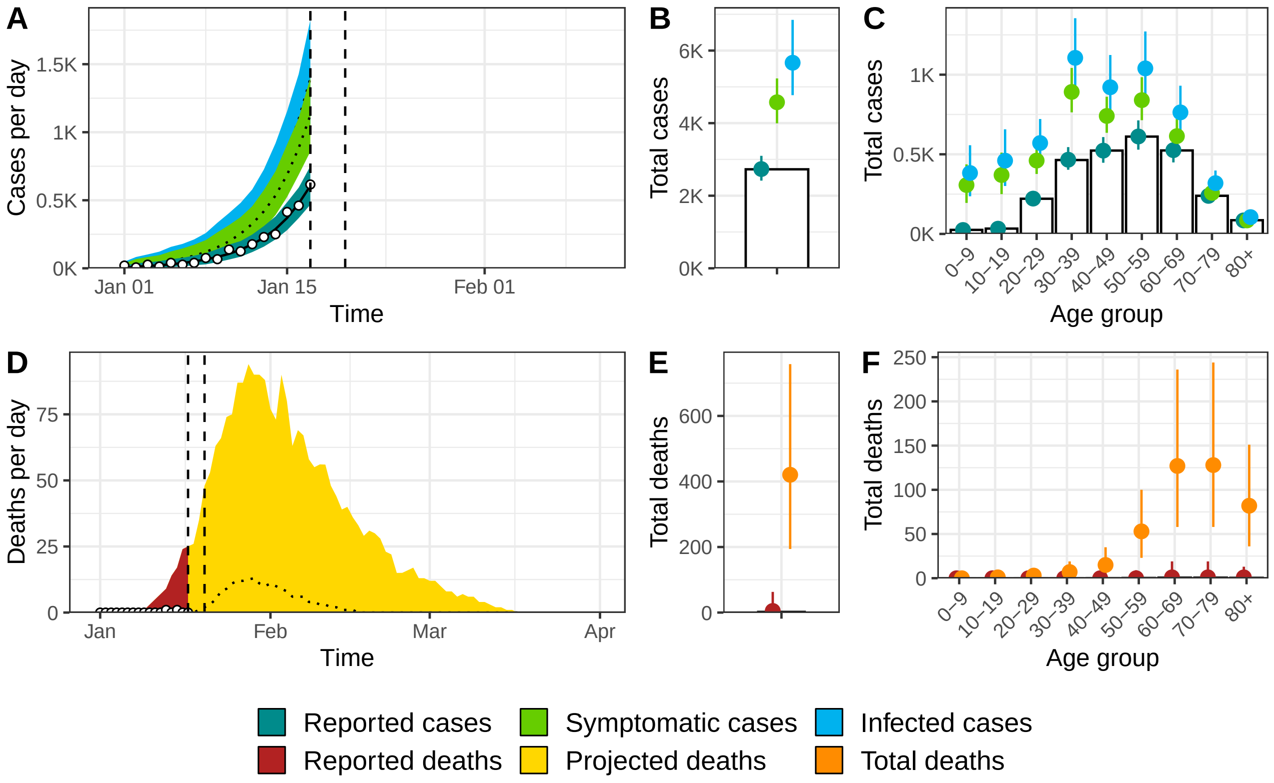

Figure S20: Model fit for Hubei, China **when the analysis is conducted until 17 January**: (A) incident cases of SARS-CoV-2 infection by date of symptom onset, (B) total cases, (C) age distribution of cases, (D) incidence of deaths, (E) total deaths and (F) age distribution of deaths. White circles and bars represent data. Lines and shaded areas or points and ranges show the posterior median and 95% credible intervals for five types of model output: reported cases, symptomatic cases, overall cases (i.e. symptomatic and asymptomatic cases), reported deaths until 17 January 2020, and overall deaths including these that will occur after this date.

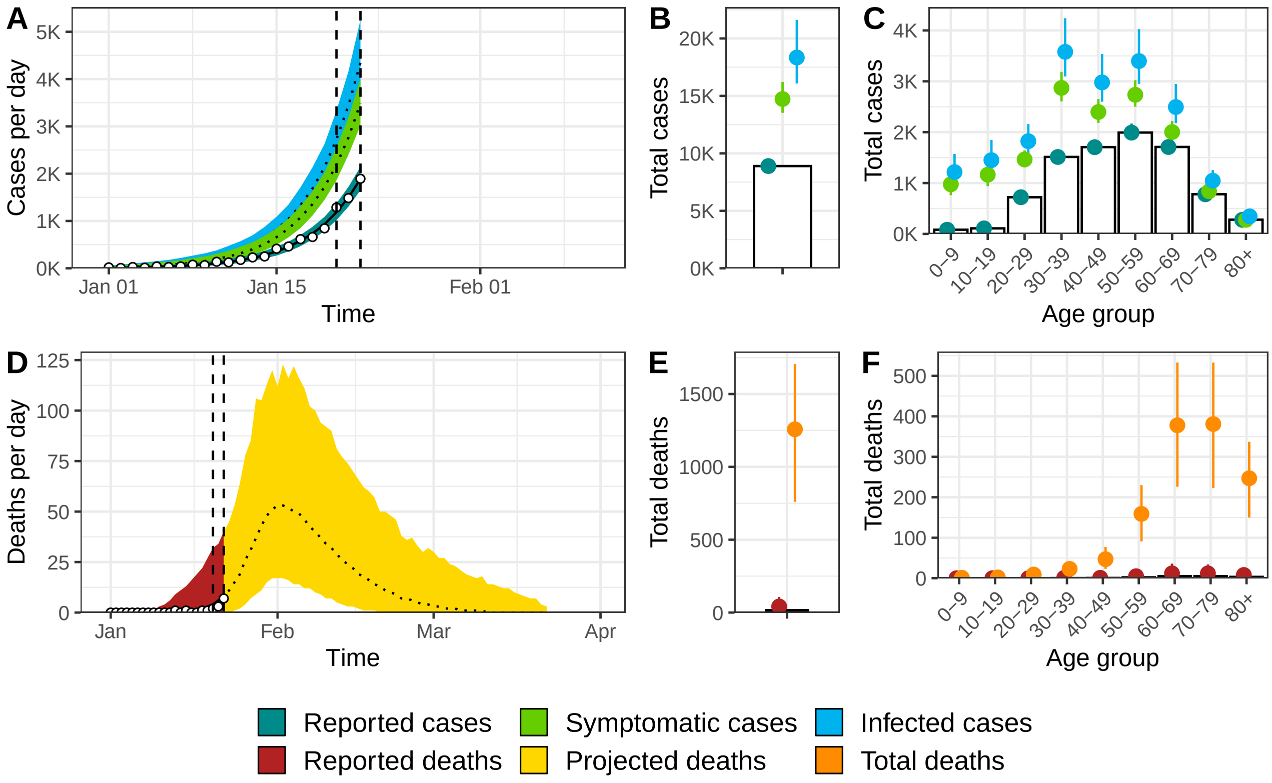

Figure S21: Model fit for Hubei, China **when the analysis is conducted until 22 January**: (A) incident cases of SARS-CoV-2 infection by date of symptom onset, (B) total cases, (C) age distribution of cases, (D) incidence of deaths, (E) total deaths and (F) age distribution of deaths. White circles and bars represent data. Lines and shaded areas or points and ranges show the posterior median and 95% credible intervals for five types of model output: reported cases, symptomatic cases, overall cases (i.e. symptomatic and asymptomatic cases), reported deaths until 27 January, and overall deaths including these that will occur after this date.

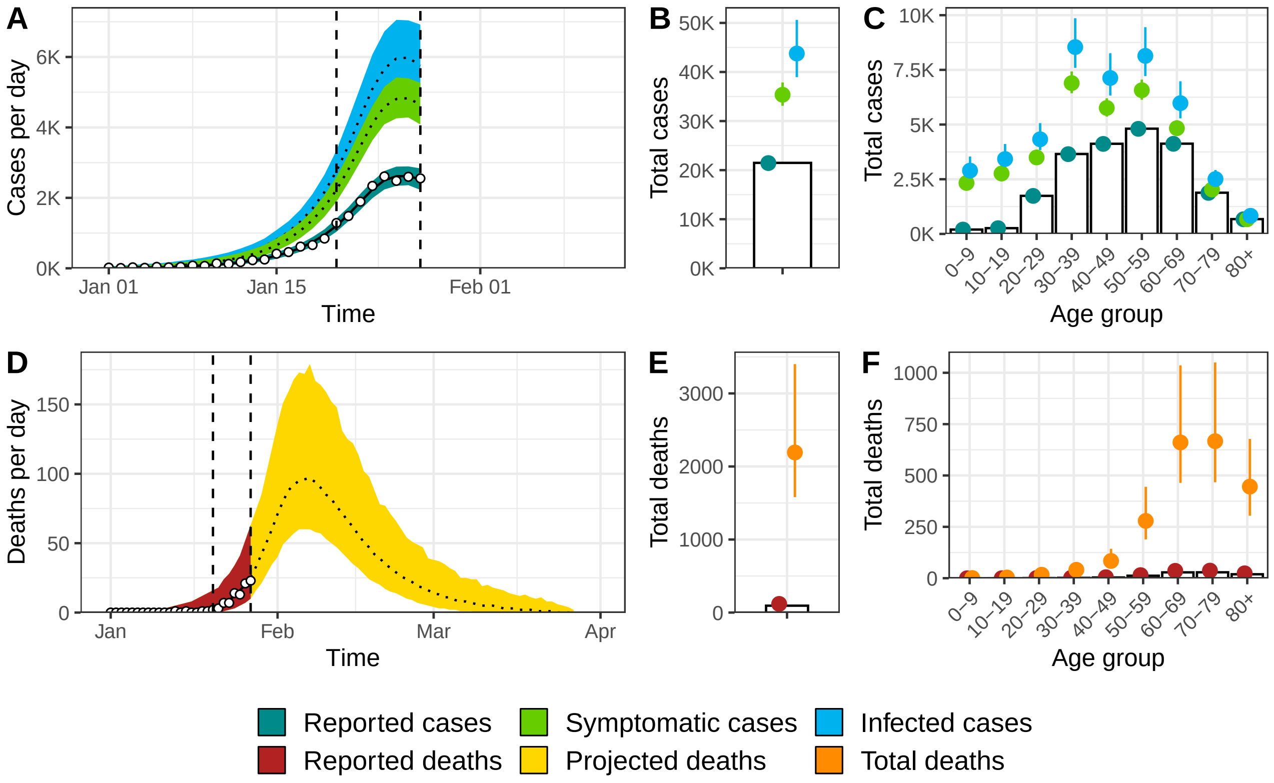

Figure S22: Model fit for Hubei, China **when the analysis is conducted until 27 January**: (A) incident cases of SARS-CoV-2 infection by date of symptom onset, (B) total cases, (C) age distribution of cases, (D) incidence of deaths, (E) total deaths and (F) age distribution of deaths. White circles and bars represent data. Lines and shaded areas or points and ranges show the posterior median and 95% credible intervals for five types of model output: reported cases, symptomatic cases, overall cases (i.e. symptomatic and asymptomatic cases), reported deaths until 27 January, and overall deaths including these that will occur after this date.

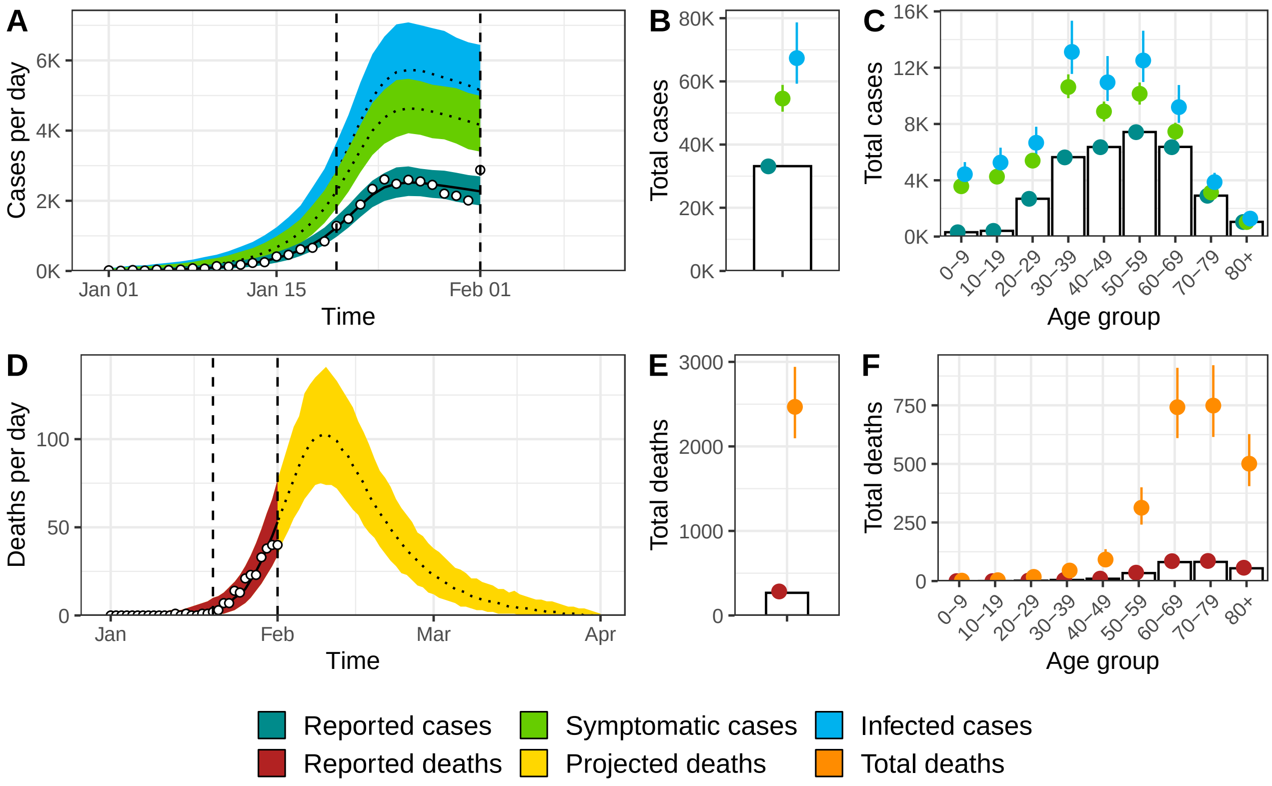

Figure S23: Model fit for Hubei, China **when the analysis is conducted until 1 February**: (A) incident cases of SARS-CoV-2 infection by date of symptom onset, (B) total cases, (C) age distribution of cases, (D) incidence of deaths, (E) total deaths and (F) age distribution of deaths. White circles and bars represent data. Lines and shaded areas or points and ranges show the posterior median and 95% credible intervals for five types of model output: reported cases, symptomatic cases, overall cases (i.e. symptomatic and asymptomatic cases), reported deaths until 27 January, and overall deaths including these that will occur after this date.

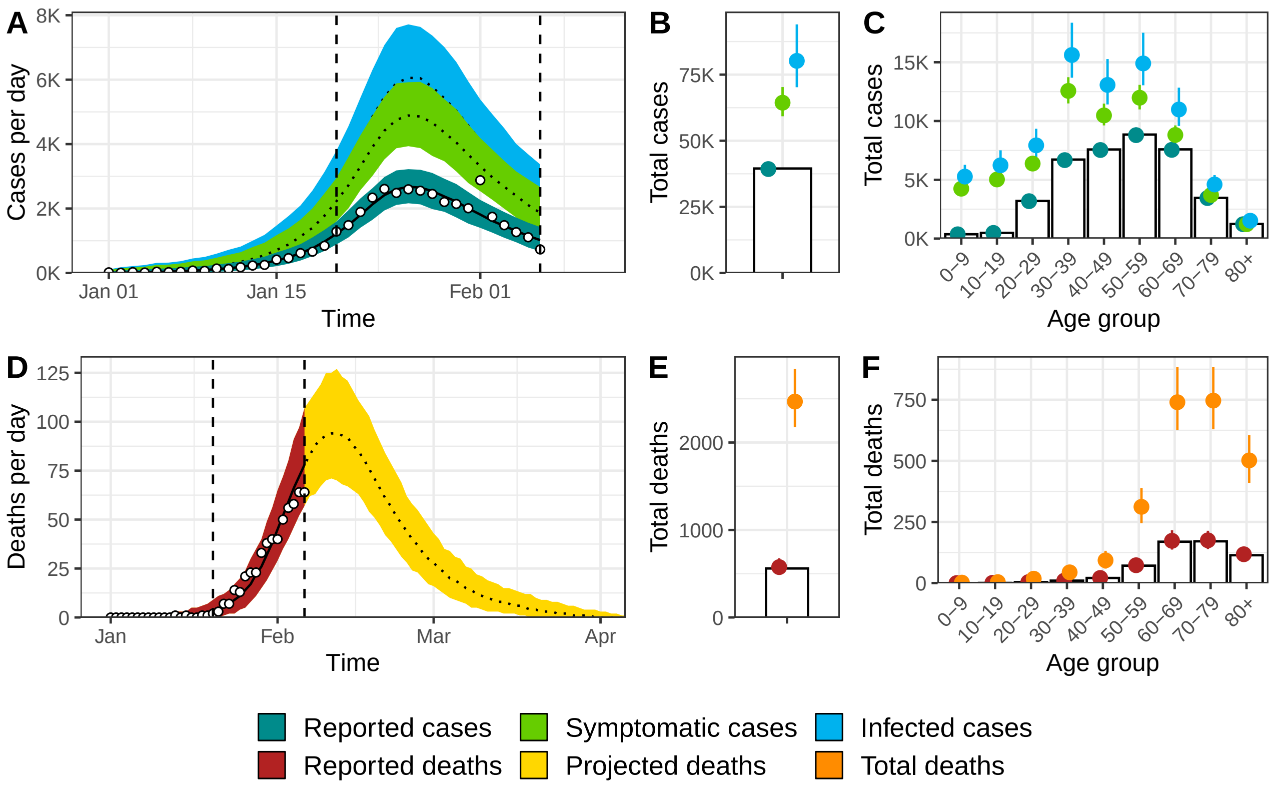

Figure S24: Model fit for Hubei, China **when the analysis is conducted until 6 February**: (A) incident cases of SARS-CoV-2 infection by date of symptom onset, (B) total cases, (C) age distribution of cases, (D) incidence of deaths, (E) total deaths and (F) age distribution of deaths. White circles and bars represent data. Lines and shaded areas or points and ranges show the posterior median and 95% credible intervals for five types of model output: reported cases, symptomatic cases, overall cases (i.e. symptomatic and asymptomatic cases), reported deaths until 6 February 2020, and overall deaths including these that will occur after this date.

6.5 Lower contribution of presymptomatic transmission

We investigated the impact of a lower contribution of presymptomatic transmission, fixed to 46% in the main analysis [11, 29, 12]. We ran two sensitivity analyses, by successively reducing the contribution of presymptomatic transmission to 30% (i.e. $q = 0.3$) and 0% (i.e. $q = 0$). As shown in Equations 9 and 11 respectively, assuming lower q decreases both the infectious period $1/\mu$ and the reduced transmission in pre- and asymptomatics κ. As a consequence, the model estimates a higher probability of infection upon contact β in order to fit the daily number of confirmed cases. When assuming $q = 0.3$, the model estimated $\beta = 96.1\%$, while both SFR (3.1% vs 2.9% in the main analysis) and IFR (3.1% vs 2.9% in the main analysis) stayed within the same range (Figure S25). When assuming $q = 0$, the model was not able to fit the number of confirmed cases anymore, even with a probability of infection upon contact $\beta = 99.5\%$, as estimated by the model (Figure S26).

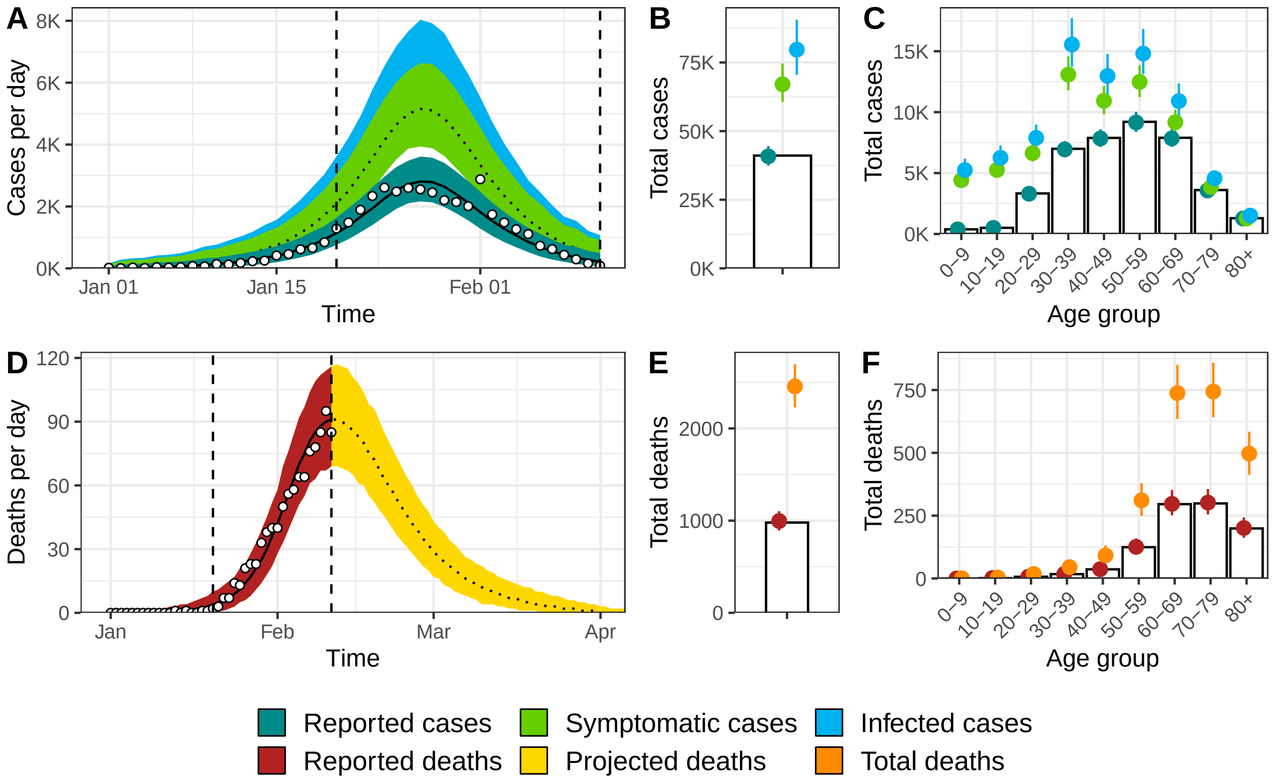

Figure S25: Model fit for Hubei, China **when the contribution of presymptomatic transmission is reduced to 30%**: (A) incident cases of SARS-CoV-2 infection by date of symptom onset, (B) total cases, (C) age distribution of cases, (D) incidence of deaths, (E) total deaths and (F) age distribution of deaths. White circles and bars represent data. Lines and shaded areas or points and ranges show the posterior median and 95% credible intervals for five types of model output: reported cases, symptomatic cases, overall cases (i.e. symptomatic and asymptomatic cases), reported deaths until 11 February 2020, and overall deaths including these that will occur after this date.

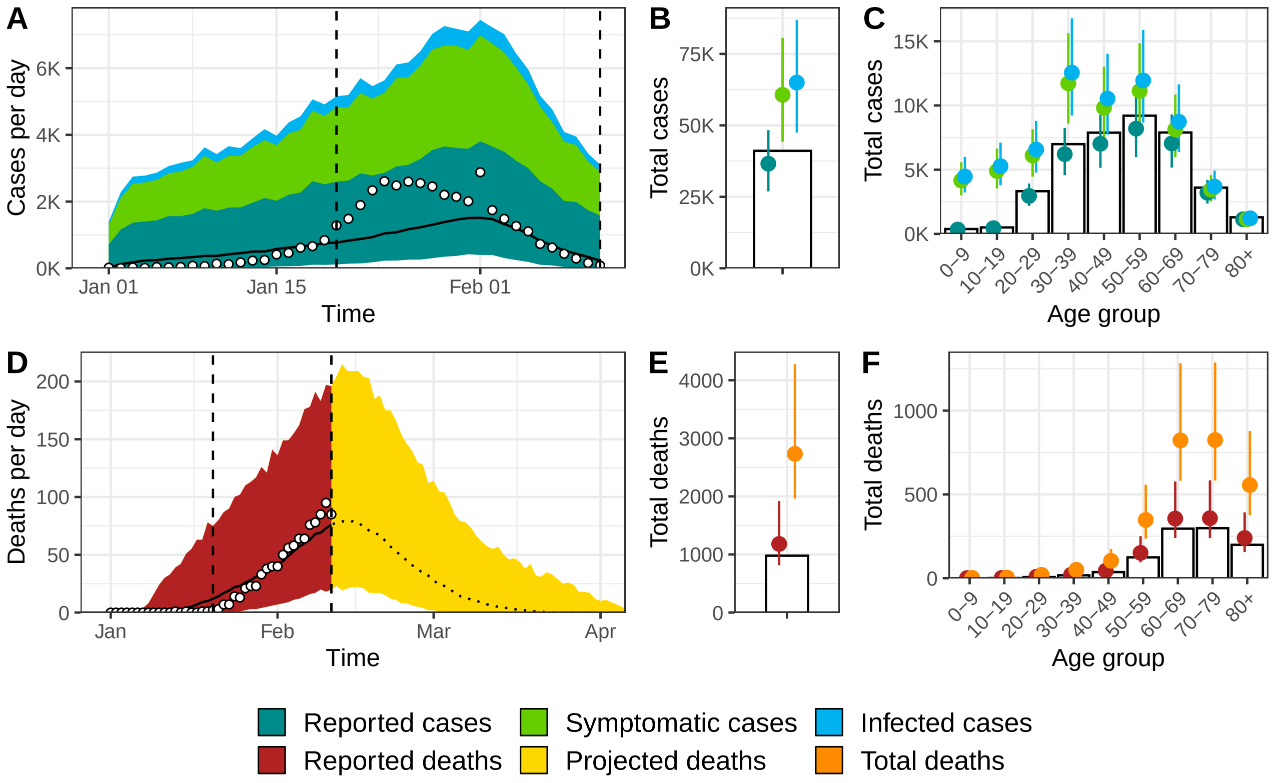

Figure S26: Model fit for Hubei, China **when the contribution of presymptomatic transmission is reduced to 0%**: (A) incident cases of SARS-CoV-2 infection by date of symptom onset, (B) total cases, (C) age distribution of cases, (D) incidence of deaths, (E) total deaths and (F) age distribution of deaths. White circles and bars represent data. Lines and shaded areas or points and ranges show the posterior median and 95% credible intervals for five types of model output: reported cases, symptomatic cases, overall cases (i.e. symptomatic and asymptomatic cases), reported deaths until 11 February 2020, and overall deaths including these that will occur after this date.

6.6 Reduced time between symptom onset and death

In the main analysis, the delay between symptom onset and death was implemented using a log-normal distribution with mean 20.2 days and standard deviation 11.6. In this sensitivity analysis, we ran the analysis in Hubei using instead a log-normal distribution with mean 15 days and standard deviation 10. This resulted in a worse fit to data and less convincing external validation (Figure S27D).

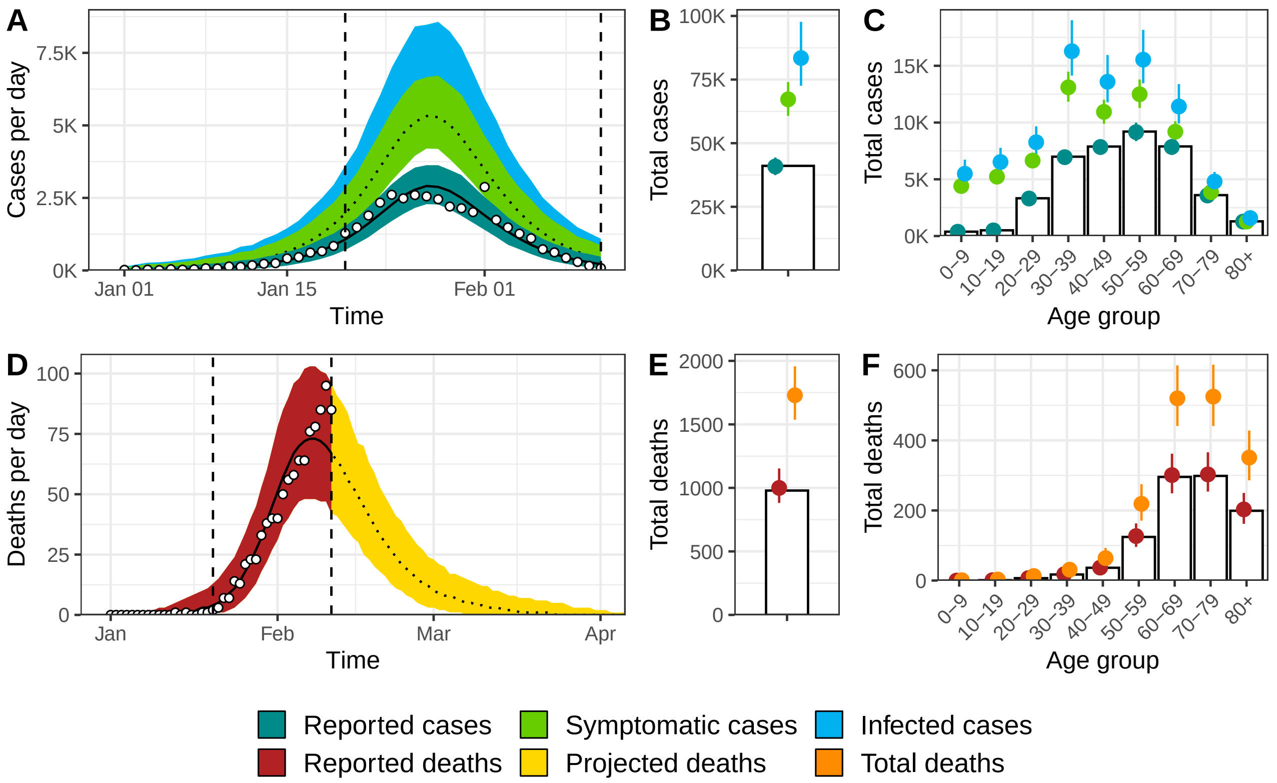

Figure S27: Model fit for Hubei, China **when the delay between onset and death is reduced to 15±10 days**: (A) incident cases of SARS-CoV-2 infection by date of symptom onset, (B) total cases, (C) age distribution of cases, (D) incidence of deaths, (E) total deaths and (F) age distribution of deaths. White circles and bars represent data. Lines and shaded areas or points and ranges show the posterior median and 95% credible intervals for five types of model output: reported cases, symptomatic cases, overall cases (i.e. symptomatic and asymptomatic cases), reported deaths until 11 February 2020, and overall deaths including these that will occur after this date.

7 Stan code

We provide here the code for the model, also available at https://github.com/jriou/covid_adjusted_cfr/. A tutorial on how to fit transmission models within Stan has been recently published and should provide guidance [31].

```
// applicable to data on cases by date of onset
functions {
real switch_eta(real t, real t1, real eta, real nu, real xi) {
return(eta+(1-eta)/(1+exp(xi*(t-t1-nu))));
}
real[] SEIR(real t,
real[] y,
real[] theta,
real[] x_r,
int[] x_i
) {
int K = x_i[1];

real tswitch = x_r[1]; // time of control measures
real dydt[(6*K)]; // SEPIAR (ignoring R) then C
real nI; // total infectious

real beta; // transmission rate
real eta; // reduction in transmission rate after quarantine
real xi; // slope of quarantine implementation
real nu; // shift of quarantine implementation
real tau_1; // infection to preclinical
real tau_2; // preclinical to symptoms (tau_1+tau_2 = incubation)
real q_P; // contribution of presymptomatics to transmission
real gt; // generation time
real mu; // infectious duration for symptomatics
real psi; // probability of symptoms
real kappa; // reduced transmissibility of preclinical and asymptomatics
real p_tswitch; // switch function
real contact[K*K]; // contact matrix, first K values, corresponds to number of contact between
  age class 1 and other classes, etc
real f_inf[K]; // force of infection
real init[K*2]; // initial values
real age_dist[K]; // age distribution of the general population
real pi; // number of cases at t0

// Estimated parameters
beta = theta[1];
eta = theta[2];
xi = theta[3];
nu = theta[4];
pi = theta[5];
psi = theta[6];

// Fixed parameters
tau_1 = x_r[2];
tau_2 = x_r[3];
q_P = x_r[4];
gt = x_r[5];

// Composite parameters
mu = (1-q_P)/(gt-1/tau_1-1/tau_2);
kappa = (q_P*tau_2*psi)/((1-q_P)*mu-(1-psi)*q_P*tau_2);

// Contact matrix
```

```
contact = x_r[6:(5+K*K)];

// Initial conditions
for(k in 1:K){
age_dist[k] = x_r[5+K*K + k];
init[k] = age_dist[k] * (1-pi);
init[K+k] = age_dist[k] * pi;
}

// Total number of infectious people
p_tswitch = switch_eta(t,tswitch,eta,nu,xi);

// Force of infection by age classes: beta * p_tswitch * sum((number of infected people by
  age + kappa*number of preclinical by age + kappa*number of asympto) / (total number of
  people by age) * (number of contact by age))
for(k in 1:K) {
f_inf[k] = beta * p_tswitch * sum((to_vector(y[(3*K+1):(4*K)])+kappa*to_vector(y[(2*K+1):(3*K)])+
  kappa*to_vector(y[(4*K+1):(5*K)]))./ to_vector(age_dist) .* to_vector(contact[(K*(k-1)+1):(k*K)]));
}

// Compartments
for (k in 1:K) {
// S: susceptible
dydt[k] = - f_inf[k] * (y[k]+init[k]);
// E: incubating (not yet infectious)
dydt[K+k] = f_inf[k] * (y[k]+init[k]) - tau_1 * (y[K+k]+init[K+k]);
// P: presymptomatic (incubating and infectious)
dydt[2*K+k] = tau_1 * (y[K+k]+init[K+k]) - tau_2 * y[2*K+k];
// I: symptomatic
dydt[3*K+k] = psi * tau_2 * y[2*K+k] - mu * y[3*K+k];
// A: asymptomatic
dydt[4*K+k] = (1-psi) * tau_2 * y[2*K+k] - mu * y[4*K+k];
// C: cumulative number of infections by date of symptom onset
dydt[5*K+k] = psi * tau_2 * y[2*K+k];
}
return(dydt);
}
}

data {
// Structure
int K; // number of age classes
vector[K] age_dist; // age distribution of the population
int pop_t; // total population
real tswitch; // time of introduction of control measures
// Controls
real t0; //starting time
int t_data; //time of first data
int S;
real ts[S]; // time bins
int inference; // 0: simulating from priors; 1: fit to data
int doprint;
// Data to fit
int D; // number of days with reported incidence
int incidence_cases[D]; // overal incidence for W weeks
int incidence_deaths[D]; // overal incidence for W weeks
int agedistr_cases[K]; // number of cases at tmax for the K age classes
int agedistr_deaths[K]; // mortality at tmax for the K age classes
// Priors
real p_beta;
real p_eta[2];
```

```
real p_pi[2];
real p_epsilon[2];
real p_rho[2];
real p_phi;
real p_xi;
real p_nu;
real p_psi[2];
// Fixed parameters
real contact[K*K]; // contact matrix
real p_q_P; // proportion of transmission that is caused by presymptomatics
real p_incubation; // incubation period
real p_preclinical; // preclinical period (part of the incubation with possible transmission)
real p_generation_time;
real p_children_trans; // relative transmissibility in children 1-10
// Fixed corrections
real p_report_80plus; // fixed ascertainment proportion for ages 80+
real p_underreport_deaths; // correction for deaths reported later
real p_underreport_cases; // correction for cases reported later
// Fixed delays
int G;
real p_gamma[G]; // from onset to death
}

transformed data {
real tau_1 = 1.0 / (p_incubation - p_preclinical);
real tau_2 = 1.0 / p_preclinical;
real q_P = p_q_P;
real gt = p_generation_time;
real x_r[5+K*K+K]; // 5 parameters + K*K contact matrix parameters + K age_dist parameters
int x_i[1] = {K};
real init[K*6] = rep_array(0.0, K*6); // initial values
real contact2[K*K] = contact;
for(i in 1:(2*K)) contact2[i] = contact[i] * p_children_trans; // lower transmissibility in children
x_r[1] = tswitch;
x_r[2] = tau_1;
x_r[3] = tau_2;
x_r[4] = q_P;
x_r[5] = gt;
x_r[6:(5+K*K)] = contact2;
for(k in 1:K) {
x_r[5+K*K+k] = age_dist[k];
}
}

parameters{
real<lower=0,upper=1> beta; // base transmission rate
real<lower=0,upper=1> eta; // reduction in transmission rate after quarantine measures
vector<lower=0,upper=1> [K] epsilon; // age-dependent mortality probability
vector<lower=0,upper=1> [K-1] raw_rho; // age-dependent reporting probability
real<lower=0, upper=1> pi; // number of cases at t0
real<lower=0> phi[2]; // variance parameters
real<lower=0,upper=1> xi_raw; // slope of quarantine implementation
real<lower=0> nu; // shift of quarantine implementation
real<lower=0,upper=1> psi; // proportion of symptomatics
}
transformed parameters {
// transformed parameters
vector[K] rho;
real xi = xi_raw+0.5;
// change of format for integrate_ode_rk45
real theta[6]; // vector of parameters
```

```
real y[S,K*6]; // raw ODE output
vector[K] comp_C[S+G];
vector[K] comp_diffC[S+G];
vector[K] comp_M[S+G];
vector[K] comp_diffM[S+G];
// outcomes
vector[D] output_incidence_cases; // overall case incidence by day
vector[D] output_incidence_deaths; // overal mortality incidence by day
simplex[K] output_agedistr_cases; // final age distribution of cases
simplex[K] output_agedistr_deaths; // final age distribution of deaths

// transformed paremeters
for(i in 1:(K-1)){
rho[i] = raw_rho[i]*p_report_80plus;
}
rho[K] = p_report_80plus; // fixed ascertainment proportion for ages 80+
// change of format for integrate_ode_rk45
theta[1:6] = {beta,eta,xi,nu,pi,psi};
// run ODE solver
y = integrate_ode_bdf(
SEIR, // ODE function
init, // initial states
t0, // t0
ts, // evaluation dates (ts)
theta, // parameters
x_r, // real data
x_i, // integer data
1.0E-10, 1.0E-10, 1.0E3); // tolerances and maximum steps
// extract and format ODE results (1.0E-9 correction to avoid negative values due to
  unprecise estimates of zeros as tolerance is 1.0E-10)
for(i in 1:S) {
comp_C[i] = (to_vector(y[i,(5*K+1):(6*K)]) + 1.0E-9) * pop_t;
comp_diffC[i] = i==1 ? comp_C[i,] : 1.0E-9*pop_t + comp_C[i,] - comp_C[i-1,]; // lagged difference
   of cumulative incidence of symptomatics
}
// Incidence and cumulative incidence after S
for(g in 1:G) {
comp_C[S+g] = comp_C[S];
comp_diffC[S+g] = rep_vector(1.0E-9,K);
}
// Mortality
for(i in 1:(S+G)) comp_diffM[i] = rep_vector(1.0E-9,K);
for(i in 1:S) for(g in 1:G) comp_diffM[i+g] += comp_diffC[i] .* epsilon * p_gamma[g];
for(i in 1:(S+G)) for(k in 1:K) comp_M[i,k] = sum(comp_diffM[1:i,k]);
// Compute outcomes
for(i in t_data:S){
output_incidence_cases[i-t_data+1] = sum(comp_diffC[i].*rho)*p_underreport_cases;
output_incidence_deaths[i-t_data+1] = sum(comp_diffM[i])*p_underreport_deaths;
}
output_agedistr_cases = (comp_C[S,].*rho) ./ sum(comp_C[S,].*rho);
output_agedistr_deaths = (comp_M[S,]) ./ sum(comp_M[S,]);
}
model {
// priors
beta ~ beta(p_beta,p_beta);
eta ~ beta(p_eta[1],p_eta[2]);
for(k in 1:K) epsilon[k] ~ beta(p_epsilon[1],p_epsilon[2]);
for(k in 1:(K-1)) raw_rho[k] ~ beta(p_rho[1],p_rho[2]);
pi ~ beta(p_pi[1],p_pi[2]);
phi ~ exponential(p_phi);
xi_raw ~ beta(p_xi,p_xi);
```

```
nu ~ exponential(p_nu);
psi ~ beta(p_psi[1],p_psi[2]);
// debug
if(doprint==1) {
print("beta: ",beta);
print("eta: ",beta);
print("epsilon: ",epsilon);
print("rho: ",rho);
print("pi: ",pi);
print("y[5,]: ",y[5,]);
print("comp_C[5,]: ",comp_C[5,]);
print("comp_diffC[5,]: ",comp_diffC[5,]);
print("comp_M[5,]: ",comp_M[5,]);
print("comp_diffM[5,]: ",comp_diffM[5,]);
}
// likelihood
if (inference!=0) {
for(i in 1:D) {
target += neg_binomial_2_lpmf( incidence_cases[i] | output_incidence_cases[i],
  output_incidence_cases[i]/phi[1]);
target += neg_binomial_2_lpmf( incidence_deaths[i] | output_incidence_deaths[i],
  output_incidence_deaths[i]/phi[2]);
}
target += multinomial_lpmf(agedistr_cases | output_agedistr_cases);
target += multinomial_lpmf(agedistr_deaths | output_agedistr_deaths);
}
}

generated quantities{
real avg_rho = sum(age_dist .* rho);
real beta2 = beta*eta;
real mu = (1-q_P)/(gt-1/tau_1-1/tau_2);
real kappa = (q_P*tau_2*psi)/((1-q_P)*mu-(1-psi)*q_P*tau_2);

int predicted_reported_incidence_symptomatic_cases[S];
real predicted_overall_incidence_symptomatic_cases[S];
real predicted_overall_incidence_all_cases[S];
int predicted_reported_incidence_deaths[S+G];
real predicted_overall_incidence_deaths[S+G];

int predicted_comp_reported_diffC[S,K];
vector[K] predicted_comp_overall_diffC[S];
vector[K] predicted_comp_overall_diffA[S];
int predicted_comp_diffM[S+G,K];

vector[K] predicted_total_reported_symptomatic_cases_by_age;
vector[K] predicted_total_overall_symptomatic_cases_by_age;
vector[K] predicted_total_overall_all_cases_by_age;
vector[K] predicted_total_overall_deaths_tmax_by_age;
vector[K] predicted_total_overall_deaths_delay_by_age;

real predicted_total_reported_symptomatic_cases;
real predicted_total_overall_symptomatic_cases;
real predicted_total_overall_all_cases;
real predicted_total_overall_deaths_tmax;
real predicted_total_overall_deaths_delay;

vector[K] cfr_A_symptomatic_by_age; //cfr by age classes, no correction of underreporting,
  no correction of time lag
vector[K] cfr_B_symptomatic_by_age; //cfr by age classes, no correction of underreporting,
correction of time lag
```

```
vector[K] cfr_C_symptomatic_by_age; //cfr by age classes, correction of underreporting,
no correction of time lag
vector[K] cfr_D_symptomatic_by_age; //cfr by age classes, correction of underreporting,
correction of time lag
vector[K] cfr_C_all_by_age; //cfr by age classes, correction of underreporting and asymptomatics,
no correction of time lag
vector[K] cfr_D_all_by_age; //cfr by age classes, correction of underreporting and asymptomatics,
correction of time lag
real cfr_A_symptomatic; //cfr by age classes, no correction of underreporting,
no correction of time lag
real cfr_B_symptomatic; //cfr by age classes, no correction of underreporting,
 correction of time lag
real cfr_C_symptomatic; //cfr by age classes, correction of underreporting,
no correction of time lag
real cfr_D_symptomatic; //cfr by age classes, correction of underreporting,
 correction of time lag
real cfr_C_all; //cfr by age classes, correction of underreporting and asymptomatics,
 no correction of time lag
real cfr_D_all; //cfr by age classes, correction of underreporting and asymptomatics,
 correction of time lag

for(i in 1:S){
predicted_reported_incidence_symptomatic_cases[i] = neg_binomial_2_rng(sum(comp_diffC[i].*rho)
  *p_underreport_cases, sum(comp_diffC[i].*rho)*p_underreport_cases/phi[1]);
predicted_overall_incidence_symptomatic_cases[i] = predicted_reported_incidence_symptomatic_cases[i] /
  p_underreport_cases / avg_rho;
predicted_overall_incidence_all_cases[i] = predicted_reported_incidence_symptomatic_cases[i] /
  p_underreport_cases / avg_rho / psi;
}
for(i in 1:(S+G)) {
predicted_reported_incidence_deaths[i] = neg_binomial_2_rng(sum(comp_diffM[i])*p_underreport_deaths,
  sum(comp_diffM[i])*p_underreport_deaths/phi[2]);
predicted_overall_incidence_deaths[i] = (1e-9+predicted_reported_incidence_deaths[i]) /
  p_underreport_deaths;
}
for(i in 1:S) {
predicted_comp_reported_diffC[i] = predicted_reported_incidence_symptomatic_cases[i] == 0 ?
  rep_array(0,K) : multinomial_rng(output_agedistr_cases,
  predicted_reported_incidence_symptomatic_cases[i]);
predicted_comp_overall_diffC[i] = to_vector(predicted_comp_reported_diffC[i]) ./ rho /
   p_underreport_cases;
predicted_comp_overall_diffA[i] = to_vector(predicted_comp_reported_diffC[i]) ./ rho * (1-psi) /
   psi / p_underreport_cases;
}
for(i in 1:(S+G)) predicted_comp_diffM[i] = predicted_reported_incidence_deaths[i] == 0 ?
   rep_array(0,K) : multinomial_rng(output_agedistr_deaths,predicted_reported_incidence_deaths[i]);
for(i in 1:K) {
predicted_total_reported_symptomatic_cases_by_age[i] = sum(predicted_comp_reported_diffC[1:S,i]) ;
predicted_total_overall_symptomatic_cases_by_age[i] = sum(predicted_comp_overall_diffC[1:S,i]);
predicted_total_overall_all_cases_by_age[i] = sum(predicted_comp_overall_diffC[1:S,i]) +
   sum(predicted_comp_overall_diffA[1:S,i]);
predicted_total_overall_deaths_tmax_by_age[i] = sum(predicted_comp_diffM[1:S,i]) /
  p_underreport_deaths;
predicted_total_overall_deaths_delay_by_age[i] = sum(predicted_comp_diffM[1:(S+G),i]) /
  p_underreport_deaths;
}
predicted_total_reported_symptomatic_cases = sum(predicted_total_reported_symptomatic_cases_by_age);
predicted_total_overall_symptomatic_cases = sum(predicted_total_overall_symptomatic_cases_by_age);
predicted_total_overall_all_cases = sum(predicted_total_overall_all_cases_by_age);
predicted_total_overall_deaths_tmax = sum(predicted_total_overall_deaths_tmax_by_age);
predicted_total_overall_deaths_delay = sum(predicted_total_overall_deaths_delay_by_age);
```

```
cfr_A_symptomatic_by_age = predicted_total_overall_deaths_tmax_by_age ./
  predicted_total_reported_symptomatic_cases_by_age;
cfr_B_symptomatic_by_age = predicted_total_overall_deaths_delay_by_age ./
  predicted_total_reported_symptomatic_cases_by_age;
cfr_C_symptomatic_by_age = predicted_total_overall_deaths_tmax_by_age ./
  predicted_total_overall_symptomatic_cases_by_age;
cfr_D_symptomatic_by_age = predicted_total_overall_deaths_delay_by_age ./
  predicted_total_overall_symptomatic_cases_by_age;

cfr_A_symptomatic = predicted_total_overall_deaths_tmax / predicted_total_reported_symptomatic_cases;
cfr_B_symptomatic = predicted_total_overall_deaths_delay / predicted_total_reported_symptomatic_cases;
cfr_C_symptomatic = predicted_total_overall_deaths_tmax / predicted_total_overall_symptomatic_cases;
cfr_D_symptomatic = predicted_total_overall_deaths_delay / predicted_total_overall_symptomatic_cases;

cfr_C_all_by_age = predicted_total_overall_deaths_tmax_by_age ./
  predicted_total_overall_all_cases_by_age;
cfr_D_all_by_age = predicted_total_overall_deaths_delay_by_age ./
  predicted_total_overall_all_cases_by_age;

cfr_C_all = predicted_total_overall_deaths_tmax / predicted_total_overall_all_cases;
cfr_D_all = predicted_total_overall_deaths_delay / predicted_total_overall_all_cases;
}
```

References

[1] info W. Age-distribution in China;. Available from: https://www.worldometers.info/demographics/china-demographics/.

[2] UNCTAD. Handbook of Statistics;. Accessed: 2020-03-22. https://unctadstat.unctad.org/EN/Index.html.

[3] Zhang, Juanjuan, Klepac, Petra, Read, M J, et al. Patterns of human social contact and contact with animals in Shanghai and China. Scientific Reports. 2019 dec;9(1):1–11.

[4] Mossong J, Hens N, Jit M, Beutels P, Auranen K, Mikolajczyk R, et al. Social Contacts and Mixing Patterns Relevant to the Spread of Infectious Diseases. PLoS Medicine. 2008 mar;5(3):e74. Available from: https://dx.plos.org/10.1371/journal.pmed.0050074.

[5] Linton NM, Kobayashi T, Yang Y, Hayashi K, Akhmetzhanov AR, Jung Sm, et al. Epidemiological characteristics of novel coronavirus infection: A statistical analysis of publicly available case data. medRxiv. 2020;.

[6] Bi Q, Wu Y, Mei S, Ye C, Zou X, Zhang Z, et al. Epidemiology and Transmission of COVID-19 in Shenzhen China: Analysis of 391 cases and 1,286 of their close contacts. Lancet Inf Dis. 2020 April;.

[7] Li Q, Guan X, Wu P, Wang X, Zhou L, Tong Y, et al. Early transmission dynamics in Wuhan, China, of novel coronavirus–infected pneumonia. New England Journal of Medicine. 2020;.

[8] Linton NM, Kobayashi T, Yang Y, Hayashi K, Akhmetzhanov AR, Jung Sm, et al. Incubation Period and Other Epidemiological Characteristics of 2019 Novel Coronavirus Infections with Right Truncation: A Statistical Analysis of Publicly Available Case Data. Journal of Clinical Medicine. 2020;9(2):538.

[9] Liu Y, Funk S, Flasche S, et al. The contribution of pre-symptomatic infection to the transmission dynamics of COVID-2019. Wellcome Open Research. 2020;5(58):58.

[10] Zhang J, Litvinova M, Wang W, Wang Y, Deng X, Chen X, et al. Evolving epidemiology of novel coronavirus diseases 2019 and possible interruption of local transmission outside Hubei Province in China: a descriptive and modeling study. medRxiv. 2020;.

[11] Ganyani T, Kremer C, Chen D, Torneri A, Faes C, Wallinga J, et al. Estimating the generation interval for COVID-19 based on symptom onset data. medRxiv. 2020;.

[12] He X, Lau EH, Wu P, Deng X, Wang J, Hao X, et al. Temporal dynamics in viral shedding and transmissibility of COVID-19. Nature medicine. 2020;p. 1–4.

[13] Linton NM, Kobayashi T, Yang Y, Hayashi K, Akhmetzhanov AR, Jung Sm, et al. Incubation Period and Other Epidemiological Characteristics of 2019 Novel Coronavirus Infections with Right Truncation: A Statistical Analysis of Publicly Available Case Data. Journal of Clinical Medicine. 2020;9(2):538.

[14] Read JM, Lessler J, Riley S, Wang S, Tan LJ, Kwok KO, et al. Social mixing patterns in rural and urban areas of southern China. Proceedings of the Royal Society B: Biological Sciences. 2014;281(1785):20140268.

[15] Prem K, Cook AR, Jit M. Projecting social contact matrices in 152 countries using contact surveys and demographic data. PLoS computational biology. 2017;13(9):e1005697.

[16] Arregui S, Aleta A, Sanz J, Moreno Y. Projecting social contact matrices to different demographic structures. PLoS computational biology. 2018;14(12):e1006638.

[17] Mossong J, Hens N, Jit M, Beutels P, Auranen K, Mikolajczyk R, et al. Social contacts and mixing patterns relevant to the spread of infectious diseases. PLoS medicine. 2008;5(3).

[18] Bundesministerium für Soziales, Gesundheit, Pflege und Konsumentenschutz. Amtliches Dashboard COVID19;. Available from: `https://info.gesundheitsministerium.at/`.

[19] The Novel Coronavirus Pneumonia Emergency Response Epidemiology Team. Vital Surveillances: The Epidemiological Characteristics of an Outbreak of 2019 Novel Coronavirus Diseases (COVID-19) — China, 2020. China CDC Weekly. 2020;2(8):113–122. Available from: `http://weekly.chinacdc.cn/en/article/id/e53946e2-c6c4-41e9-9a9b-fea8db1a8f51`.

[20] Rami Krispin. R package 'Coronavirus'; 2020. Accessed: 2020-05-15. `https://ramikrispin.github.io/coronavirus/`.

[21] Presidenza del Consiglio dei Ministri - Dipartimento della Protezione Civile. GitHub repository on COVID-19;. Accessed: 2020-04-29. `https://github.com/pcm-dpc`.

[22] Istituto Superiore di Sanita. EPIDEMIA COVID-19, Aggiornamento nazionale (appendice), 28 aprile 2020 – ore 16:00;.

[23] Robert Koch Institute. Epidemiological Situation in Germany;. Available from: `https://corona.rki.de`.

[24] Ministerio de Sanidad. Actualización n° 89. Enfermedad por el coronavirus (COVID-19). 28.04.2020;.

[25] Federal office of Public Health. Personal communication from Federal office of Public Healths (FOPH), Switzerland, through SSPHplus website;.

[26] Carpenter B, Gelman A, Hoffman MD, Lee D, Goodrich B, Betancourt M, et al. Stan: A probabilistic programming language. Journal of statistical software. 2017;76(1).

[27] Anderson RM, Anderson B, May RM. Infectious diseases of humans: dynamics and control. Oxford university press; 1992.

[28] Svensson Å. A note on generation times in epidemic models. Mathematical biosciences. 2007;208(1):300–311.

[29] Liu T, Hu J, Kang M, Lin L, Zhong H, Xiao J, et al.. Transmission dynamics of 2019 novel coronavirus (2019-nCoV); 2020. Available from: `https://ssrn.com/abstract=3526307`.

[30] Buitrago-Garcia DC, Egli-Gany D, Counotte MJ, Hossmann S, Imeri H, Salanti G, et al. The role of asymptomatic SARS-CoV-2 infections: rapid living systematic review and meta-analysis. medRxiv. 2020;.

[31] Grinsztajn L, Semenova E, Margossian CC, Riou J. Bayesian workflow for disease transmission modeling in Stan; 2020. Accessed: 2020-05-15. `https://mc-stan.org/users/documentation/case-studies/boarding_school_case_study.html`.
