# Supplementary material for: Estimation of SARS-CoV-2 mortality during the early stages of an epidemic: A modeling study in Hubei, China, and six regions in Europe
Source: PLoS Med. 2020 Jul 28;17(7):e1003189. doi: 10.1371/journal.pmed.1003189 (PMC7386608; doi:10.1371/journal.pmed.1003189)
Supplement: S2 Text — Reporting of model developing and validating according to the TRIPOD Checklist for Prediction Model Development. TRIPOD, transparent reporting of a multivariable prediction model for individual prognosis or diagnosis. (DOCX) [file pmed.1003189.s002.docx]

TRIPOD Checklist: Prediction Model Development

| **Section/Topic** | **Item** | **Checklist Item** | **Section** |
| --- | --- | --- | --- |
| **Title and abstract** |  |  |  |
| Title | 1 | Identify the study as developing and/or validating a multivariable prediction model, the target population, and the outcome to be predicted. | Title |
| Abstract | 2 | Provide a summary of objectives, study design, setting, participants, sample size, predictors, outcome, statistical analysis, results, and conclusions. | Abstract |
| **Introduction** |  |  |  |
| Background and objectives | 3a | Explain the medical context (including whether diagnostic or prognostic) and rationale for developing or validating the multivariable prediction model, including references to existing models. | Second paragraph, section “Introduction”, p.3 |
|  | 3b | Specify the objectives, including whether the study describes the development or validation of the model or both. | Last paragraph, section “Introduction” |
| **Methods** |  |  |  |
| Source of data | 4a | Describe the study design or source of data (e.g., randomized trial, cohort, or registry data), separately for the development and validation data sets, if applicable. | Subsections “Setting and data, Hubei province, China” and “Setting and data, six geographic locations in Europe”, in section “Method” and S1 File Section 1 |
|  | 4b | Specify the key study dates, including start of accrual; end of accrual; and, if applicable, end of follow-up. |  |
| Participants | 5a | Specify key elements of the study setting (e.g., primary care, secondary care, general population) including number and location of centres. |  |
|  | 5b | Describe eligibility criteria for participants. | NA |
|  | 5c | Give details of treatments received, if relevant. | Not relevant |
| Outcome | 6a | Clearly define the outcome that is predicted by the prediction model, including how and when assessed. | Subsection “Age-structured model of SARS-CoV-2 transmission and mortality” in section “Method” |
|  | 6b | Report any actions to blind assessment of the outcome to be predicted. | NA |
| Predictors | 7a | Clearly define all predictors used in developing or validating the multivariable prediction model, including how and when they were measured. | Subsection “Age-structured model of SARS-CoV-2 transmission and mortality” in section “Method” |
|  | 7b | Report any actions to blind assessment of predictors for the outcome and other predictors. | NA |
| Sample size | 8 | Explain how the study size was arrived at. | Based on available data, S1 File Section 1 |
| Missing data | 9 | Describe how missing data were handled (e.g., complete-case analysis, single imputation, multiple imputation) with details of any imputation method. | S1 File section 1 |
| Statistical analysis methods | 10a | Describe how predictors were handled in the analyses. | Subsection “Age-structured model of SARS-CoV-2 transmission and mortality” in section “Method” |
|  | 10b | Specify type of model, all model-building procedures (including any predictor selection), and method for internal validation. |  |
|  | 10d | Specify all measures used to assess model performance and, if relevant, to compare multiple models. | S1 File section 3.6 |
| Risk groups | 11 | Provide details on how risk groups were created, if done. | Subsection “Age-structured model of SARS-CoV-2 transmission and mortality” in section “Method” |
| **Results** |  |  |  |
| Participants | 13a | Describe the flow of participants through the study, including the number of participants with and without the outcome and, if applicable, a summary of the follow-up time. A diagram may be helpful. | NA, cross sectional (surveillance) data |
|  | 13b | Describe the characteristics of the participants (basic demographics, clinical features, available predictors), including the number of participants with missing data for predictors and outcome. | Demographics (age) are provided in the section “Results” |
| Model development | 14a | Specify the number of participants and outcome events in each analysis. | Section “Results” |
|  | 14b | If done, report the unadjusted association between each candidate predictor and outcome. | NA |
| Model  specification | 15a | Present the full prediction model to allow predictions for individuals (i.e., all regression coefficients, and model intercept or baseline survival at a given time point). | Section “Results” and S1 File section 5 |
|  | 15b | Explain how to the use the prediction model. | NA |
| Model performance | 16 | Report performance measures (with CIs) for the prediction model. | Fig2. Model fit for Hubei, China, and S1 File Fig S10-S15. Model fit for six areas in Europe |
| **Discussion** |  |  |  |
| Limitations | 18 | Discuss any limitations of the study (such as nonrepresentative sample, few events per predictor, missing data). | Section “Discussion”, subsection “Strengths and limitations” |
| Interpretation | 19b | Give an overall interpretation of the results, considering objectives, limitations, and results from similar studies, and other relevant evidence. | Section “Discussion”, subsection “Interpretation and implications” |
| Implications | 20 | Discuss the potential clinical use of the model and implications for future research. |  |
| **Other information** |  |  |  |
| Supplementary information | 21 | Provide information about the availability of supplementary resources, such as study protocol, Web calculator, and data sets. | Provided: <https://github.com/jriou/covid_adjusted_cfr> |
| Funding | 22 | Give the source of funding and the role of the funders for the present study. | Provided, section “Funding” and “Authors’ contributions” |
